# Three new Russula species in sect. Ingratae (Russulales, Basidiomycota) from southern China

**DOI:** 10.3897/mycokeys.84.68750

**Published:** 2021-11-08

**Authors:** Guo-Jie Li, Shou-Mian Li*, Bart Buyck, Shi-Yi Zhao, Xue-Jiao Xie, Lu-Yao Shi, Chun-Ying Deng, Qing-Feng Meng, Qi-Biao Sun, Jun-Qing Yan, Jing Wang, Ming Li

**Affiliations:** 1 Key Laboratory of Vegetable Germplasm Innovation and Utilization of Hebei, Collaborative Innovation Center of Vegetable, College of Horticulture, Hebei Agricultural University, No 2596 South Lekai Rd, Lianchi District, Baoding 071001, Hebei Province, China Hebei Agricultural University Baoding China; 2 Institut de Systématique, Ecologie et Biodiversité (ISYEB), Muséum national d’Histoire naturelle, CNRS, Sorbonne Université, EPHE, 57 rue Cuvier, CP 39, 75005 Paris, France Sorbonne Université Paris France; 3 Institute of Biology, Guizhou Academy of Sciences, No 1 Shanxi Rd, Yunyan District, Guiyang 550001, Guizhou Province, China Institute of Biology, Guizhou Academy of sciences Guiyang China; 4 School of Public Health, Zunyi Medical University, No.201 Dalian Road, Huichuan District, Zunyi 563003, Guizhou Province, China Zunyi Medicinal University Zunyi China; 5 College of Pharmacy and Life Science, Jiujiang University, No 320 East Xunyang Rd, Xunyang District, Jiujiang 332000, Jiangxi Province, China Jiujiang University Jiujiang China; 6 Jiangxi Fungal Resources Laboratory of Protection and Utilization, College of Bioscience and Bioengineering, Jiangxi Agricultural University, No1101 Zhimin Rd, Qingshanhu District, Nanchang 330045, Jiangxi Province, China Jiangxi Agricultural University Nanchang China

**Keywords:** Agaricomycetes, ITS, morphology, phylogeny, Russulaceae, taxonomy

## Abstract

Three new species of Russulasection Ingratae, found in Guizhou and Jiangsu Provinces, southern China, are proposed: *R.straminella*, *R.subpectinatoides* and *R.succinea*. Photographs, line drawings and detailed morphological descriptions for these species are provided with comparisons against closely-related taxa. Phylogenetic analysis of the internal transcribed spacer (ITS) region supported the recognition of these specimens as new species. Additionally, *R.indocatillus* is reported for the first time from China and morphological and phylogenetic data are provided for the Chinese specimens.

## Introduction

*Russula* Pers. is a widespread genus that contains at least 2000, but possibly as many as 3000 species worldwide (Li et al. 2019; Adamčík et al. 2019; He et al. 2019). Members of this genus form symbiotic relationships with a diversity of plant species in broad-leaved and coniferous forests, scrubland and meadows. The brightly tinged pileus, abundant sphaerocytes responsible for the fragile gills and stipe, amyloid spore ornamentation, gleocystidia staining in sulpho-aldehydes, lack of clamp connections and absence of a ramifying lactifer system ending in pseudocystidia are the main morphological features of this genus (Li et al. 2015a; Buyck et al. 2018; Looney et al. 2018). Due to frequent convergence or extreme plasticity of morphological features, precise identification of *Russula* species is difficult and establishing accurate taxonomy is challenging (Miller and Buyck 2002; Bazzicalupo et al. 2017).

Russulasect. Ingratae Quél. is characterised by tawny, ochraceous or ashy-grey to dark brown pileus with tuberculate striate margin, acute to subacute equal lamellae, flesh with a distinct fetid, spermatic or waxy odour, or like bitter almonds, cream-coloured spore print, spores partly showing inamyloid reaction in the suprahilar area, small- to medium-sized unicellular pileocystidia and articulated and branched hyphal ends in the pileipellis (Shaffer 1972; Romagnesi 1985; Sarnari 1998). The combination of these characters makes this section one of the more easily distinguishable groups in the Russula subgenus Heterophyllidiae Romagn. Recent multi-locus phylogenetic studies indicated that this morphologically well-defined group corresponded to the earlier subsections, *Foetentinae*, *Pectinatinae* and *Subvelatae*, representing a natural, well-supported monophyletic clade in phylogenetic topology of the northern temperate region (Looney et al. 2016; Buyck et al. 2018). The other easily distinguishable groups of subgenus Heterophyllidiae include subsections *Amoeninae*, *Virescentinae* and *Substriatinae*. Phylogenetic analyses also indicated it is more difficult to match a field aspect with a single monophyletic lineage (Wang et al. 2019; Deng et al. 2020; Wisitrassameewong et al. 2020).

Compared with Europe (Romagnesi 1985; Sarnari 1998), detailed analyses of Russula sect. Ingratae in Asia began relatively late. In southern China, several species were previously misidentified, based on morphological characters, with European or North American names, such as *R.foetens* Pers., *R.grata* Britzelm. (= *R.laurocerasi* Melzer) and *R.pectinatoides* Peck (Song et al. 2007; Li 2014). Rapid progress has been made in the past two decades, resulting in 15 new *Russula* species in Asian *Ingratae*, based on modern phylogenetic methods: *R.abbotensis* K. Das & J.R. Sharma, *R.ahmadii* Jabeen et al., *R.arunii* S. Paloi et al., *R.catillus* H. Lee et al., *R.dubdiana* K. Das et al., *R.foetentoides* Razaq et al., *R.gelatinosa* Y. Song & L.H. Qiu, *R.indocatillus* Ghosh et al., *R.natarajanii* K. Das et al., *R.obscuricolor* K. Das et al., *R.pseudocatillus* F. Yuan & Y. Song, *R.pseudopectinatoides* G.J. Li & H.A. Wen, *R.rufobasalis* Y. Song & L.H. Qiu, *R.subpunctipes* J. Song and *R.tsokae* K. Das et al. These new species were originally described from East Asia and the adjacent Himalayan area (Das et al. 2006, 2010, 2013, 2017; Razaq et al. 2014; Li et al. 2015b; Jabeen et al. 2017; Lee et al. 2017; Song et al. 2018, 2020; Ghosh et al. 2020). The initial sequence data have supported the valid recognition of *R.punctipes* Singer and *R.senecis* Imai, but are still lacking for *R.guangdongensis* Z.S. Bi & T.H. Li and *R.periglypta* Berk & Broome (Lee et al. 2017; Song et al. 2018). Recent rDNA ITS phylogenetic analyses of R.sect. Ingratae in the Northern Hemisphere showed numerous unknown taxa and constant misidentifications of species in this group (Avis 2012; Melera et al. 2017; Park et al. 2017).

The importance of precise identification of Russula spp. in sect. Ingratae also comes from their economic value as several species are commonly sold as edible fungi in markets of southern China under the local name “You-la-gu (oily, acrid mushroom)”. Several species of R. sect. Ingratae may cause gastrointestinal problems if not properly pre-cooked (Dai et al. 2010; Bau et al. 2014; Chen et al. 2016). During recent years, several field investigations have been carried out on campuses and, in parks, natural reserves and wild mushroom markets of south-western China to unveil the species diversity of sect. Ingratae in this region. A number of *Russula* taxa have been discovered as new to science, based on morphological and molecular phylogenetic evidence, of which three members of R.sect. Ingratae are described and illustrated herein. Additionally, we report *R.indocatillus* as a new record for China.

## Materials and methods

### Morphological analyses

Specimens were collected in Guizhou, Jiangxi and Jiangsu Provinces from July to September in 2017 and 2018. The majority of the samplings are from Guizhou Province of south-western China. This mountainous Province lies in the eastern end of the Yungui Plateau. This region has a humid subtropical monsoon climate and is mostly covered by subtropical evergreen forests (Editorial Board of Vegetation in China 1980; Chen et al. 2020). Each of the specimens was collected from different patches of forest to avoid duplications from a single mycelium. Photographs of fresh basidiocarps were taken using a Canon Powershot G1 X Mark II digital camera in the field. Macroscopic characters were recorded at the same time under daylight. The colour codes and names from Ridgway (1912) were employed in descriptions. Specimen desiccation was accomplished in a Fruit FD-770C food dryer at a constant temperature of 65 °C over 12 h. Small tissue pieces of lamellae and pileipellis for microscopic observations were taken from dried specimens, sectioned by hand with a Dorco razor blade and rehydrated in 5% potassium hydroxide (KOH). Microscopical characters were observed using a Nikon Eclipse Ci-L photon microscope and Olympus BH2 with a drawing tube. Staining of basidiospores, mycelia and cystidia were performed by chemical reaction with Melzer’s Reagent and sulphovanillin (SV). Measurements and line drawings of basidiospores (exclusive of apiculus and spore ornamentation) and elements in hymenium, pileipellis and stipitipellis were executed from microphotographs taken at 1600× magnification with a Cossim U3CMOS14000 camera. A JSM-IT300 cold-field scanning electron microscope was used for examination of basidiospore ornamentation. At least 20 observation data were employed for each morphological character of every analysed collection. The format, α/β/γ, represented the numbers of basidiospores, basidiocarps and specimens that were measured microscopically. For those basidiospore dimensions, these were indicated as (*a*–) *b*–*c* (–*d*), the extremes of the measured values (*a* and *d*) are displayed in brackets. The values of *b* and *c* are 5^th^ and 95^th^ percentiles when observed readings were arranged from small to large. Q is the ratio of basidiospore length to width. The **Q** in bold is the mean value of Q plus or minus standard deviation. The pileipellis was vertically sectioned at the edge and centre of the pileus. Shapes and sizes of basidia, cystidia and hypha were observed, measured and illustrated. For other details on microscopic observation and measurement, see Li (2014) and Adamčík et al. (2019). Exsiccatae of these new species are preserved in the Macrofungus Section, Mycological Herbarium of Guizhou Academy of Sciences (HGAS-MF), Herbarium of Hebei Agricultural University (HBAU) and Herbarium of Fungi, Jiangxi Agricultural University (HFJAU).

### DNA extraction, polymerase chain reaction (PCR) and sequencing

Tissue samples from dried specimens were ground in centrifuge tubes using abrasive rods attached to an electric drill. DNA extractions were performed using a modified CTAB method as in Li (2014). PCR reactions were carried out in a Dragonlab TC1000-G 96-well thermocycler. Sequences in the ITS region were amplified with primers ITS5 and ITS4 (White et al. 1990) using the reaction conditions of Li et al. (2019). PCR products were separated by electrophoresis on 1.2% agarose gels and stained with Biotium GelRed. The concentrations of extracted DNA and PCR products were determined by a ThermoFisher Scientific NanoDrop One spectrophotometer. Nucleotide concentration > 50 ng/μl was used as the criterion of a qualified PCR product for Sanger sequencing by GENEWIZ Inc. An ABI 3730XL DNA analyser and an Applied Biosystems Sanger sequencing kit were used following manufacturer’s procedures by Biomed Gene Technology Company (Beijing, China).

### Phylogenetic analyses

**Figure 1.**
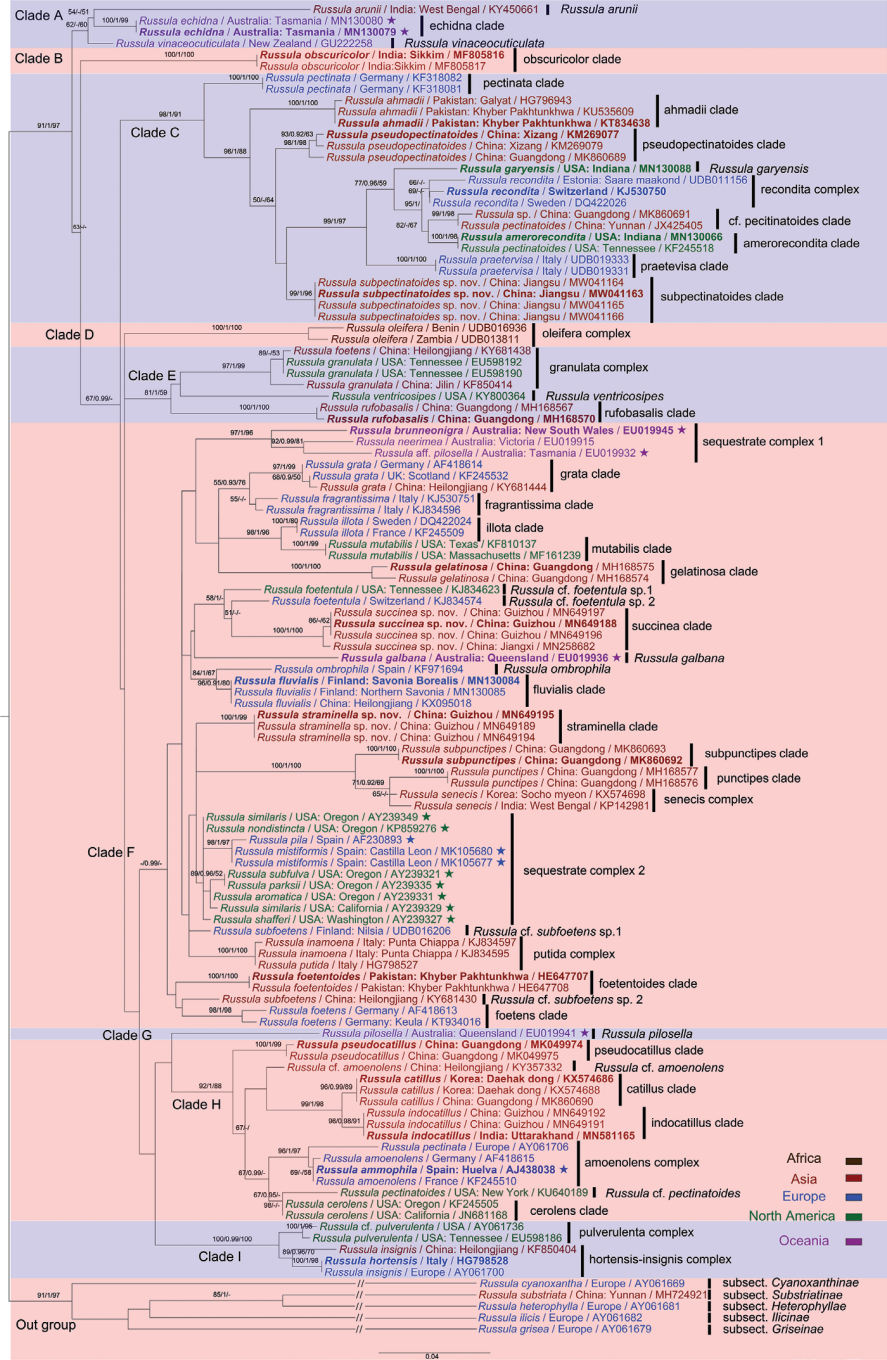
Phylogenetic tree generated from Bayesian analysis of ITS sequences. Main clades corresponding to subsections of sect. Ingratae are indicated in colour blocks. Holotypes of the new species are shown in bold. Values of posterior probabilities (PP) of MrBayes (≥ 0.9) and bootstraps of ML and MP analyses (≥ 50) are presented above the nodes as (MLBS/PP/MPBS).

Bidirectional sequencing results were assembled with MegAlign in DNAStar LaserGene 7.1 (https://www.dnastar.com). Low quality nucleotide sites at both ends of the sequences were trimmed. All new sequences from this study were deposited in GenBank (http://www.ncbi.nlm.nih.gov/nuccore/). The BLAST algorithm was used to search the similar sequences and for the new species. Table 1 contains closely matched ITS sequences of the new species (percent identities over 97%) retrieved from GenBank and UNITE (https://unite.ut.ee/) databases. Sampling for the phylogenetic backbone of Russulasection Ingratae referred to Melera et al. (2017), Park et al. (2017) and Song et al. (2018). These sequences were combined with those of the new species and aligned in Mafft 7.428 with L-INS-I strategy applied (Nakamura et al. 2018). Five sequences from species of the other sections of Russula subgenus Heterophyllidiae, *R.cyanoxantha* (Schaeff.) Fr., *R.grisea* Fr., *R.heterophylla* (Fr.) Fr., *R.ilicis* Romagn. and *R.substriata* J. Wang et al., were chosen as out-group. The matrix file was manually optimised using BioEdit 7.0.5 (Hall 1999) and deposited in TreeBASE repository with study ID S28207 (http://purl.org/phylo/treebase/phylows/study/TB2:S28207?x-access-code=cda6b439c0eada24d5199bc264971fb5&format=html). Phylogenetic analyses were executed using Bayesian Inference (BI), Maximum Likelihood (ML) and Maximum Parsimony (MP) methods. Bayesian analysis was performed in MrBayes 3.2.7a (Ronquist et al. 2012). Best evolutionary model selection was carried out with MrModeltest 2.4 operated on PAUP* 4.0a165 through Akaike’s Information Criteria (AIC) calculation (Nylander 2004). The calculation of posterior probabilities (PP) parameters was performed through the Markov chain Monte Carlo (MCMC) algorithm. The sampling frequency of the trees was set as every 100^th^ generation. One cold and three hot Markov chains were run for 2 ´ 10^6^ generations. The analysis ceased when the average standard deviation was maintained below 0.01. A percentage of 25% trees were discarded as burn-in before the construction of the 50% majority rule consensus tree. MP analysis was conducted in PAUP* 4.0a167 (Swofford 2004). The tree bisection-reconstruction (TBR) was carried out with a heuristic search. A total of 1000 replicates were set for bootstrap support (Felsenstein 1985). The setting of maxtrees was 5000. Branches collapsed when minimum length was zero. A Kishino-Hasegawa (KH) test (Kishino and Hasegawa 1989) was executed to determine whether trees were significantly different. The consistency index (CI), homoplasy index (HI), retention index (RI), rescaled consistency index (RC) and tree length (TL) were performed in MP analysis. ML analysis was performed in raxmlGUI 1.5b3 with 1000 replicates (Silvestro and Michalak 2012). Trees were displayed and exported in FigTree 1.4.4 (http://tree.bio.ed.ac.uk/software/figtree/). Names of species in Fig. 1 and Table 1 were cited from source databanks. Definitions for clades and complexes were also presented in Fig. 1.

**Table 1.** The species, specimens and GenBank accession numbers of ITS sequences analysed in this study.

Species	Specimen No.	Origin	GenBank accession	Reference
Russulaaff.pilosella	MEL H4784	Australia: Tasmania	EU019932	Lebel and Tonkin (2007)
*R.ahmadii*	LAH 35004	Pakistan: Khyber Pakhtunkhwa	KT834638	Jabeen et al. (2017)
	LAH 18081013	Pakistan: Khyber Pakhtunkhwa	KU535609	Jabeen et al. (2017)
	SB138	Pakistan	HG796943	Jabeen (2016)
*R.amerorecondita*	F PGA17-017	USA: Indiana	MN130066	Adamčík et al. (2019)
*R.ammophila*	MA-Fungi 51165	Spain: Huelva	AJ438038	Vidal et al. (2002)
*R.amoenolens*	TUB nl27.9.95.6	Germany	AF418615	Eberhardt (2002)
	MICH 12838	France	KF245510	–
R.cf.amoenolens	HMJAU37317	China: Heilongjiang	KY357332	Liu et al. (2017)
*R.aromatica*	PNW 5607	USA: Oregon	AY239331	–
*R.arunii*	CUH AM261	India: West Bengal	KY450661	Crous et al. (2017)
*R.brunneonigra*	DAR H5813	Australia: New South Wales	EU019945	Lebel and Tonkin (2007)
*R.catillus*	SFC 20120827-01	Korea: Daehak-dong	KX574686	Lee et al. (2017)
	SFC 20120919-35	Korea: Daehak-dong	KX574688	Lee et al. (2017)
	LHJ150915-19	China: Guangdong	MK860690	–
*R.cerolens*	OSC 76727	USA: Oregon	KF245505	–
	F 36	USA: California	JN681168	–
R.cf.amoenolens	MICH12838	France	KF245510	–
R.cf.pulverulenta	NYBG 4-1144IS79	USA	AY061736	Miller and Buyck (2002)
*R.cyanoxantha*	PC SM/BB 5	Europe	AY061669	Miller and Buyck (2002)
*R.echidna*	HO 593336	Australia: Tasmania	MN130079	Adamčík et al. (2019)
	HO 593337	Australia: Tasmania	MN130080	Adamčík et al. (2019)
*R.fluvialis*	KUO JR8666	Finland: Savonia Borealis	MN130084	Adamčík et al. (2019)
	KUO JR8313	Finland: Northern Savonia	MN130085	Adamčík et al. (2019)
	HMJAU 32234	China: Heilongjiang	KX095018	–
*R.foetens*	TUB hue124	Germany	AF418613	Eberhardt (2002)
	GENT FH-12-277	Germany: Keula	KT934016	Looney et al. (2016)
	HMJAU38004	China: Heilongjiang	KY681438	Liu et al. (2017)
*R.foetentoides*	LAH 04081023	Pakistan: Khyber Pakhtunkhwa	HE647707	Razaq et al. (2014)
	LAH 13081034	Pakistan: Khyber Pakhtunkhwa	HE647708	Razaq et al. (2014)
*R.foetentula*	156	USA: Tennessee	KJ834623	Melera et al. (2017)
	128	Switzerland	KJ834574	Melera et al. (2017)
*R.fragrantissima*	98	Italy	KJ530751	Melera et al. (2017)
	108	Italy	KJ834596	Melera et al. (2017)
*R.galbana*	BRIT13425	Australia: Queensland	EU019936	Lebel and Tonkin (2007)
*R.garyensis*	F PGA17-008	USA: Indiana	MN130088	Adamčík et al. (2019)
*R.gelatinosa*	K 16053119	China: Guangdong	MH168574	Song et al. (2018)
	K 15052626	China: Guangdong	MH168575	Song et al. (2018)
*R.granulata*	PC BB2004-226	USA: Tennessee	EU598192	–
	PC BB2004-225	USA: Tennessee	EU598190	–
	HMAS252604	China: Jilin	KF850414	Li (2014)
*R.grata*	E 00290534	UK: Scotland	KF245532	–
	TUB nl1348	Germany	AF418614	Eberhardt (2002)
	HMJAU38008	China: Heilongjiang	KY681444	Liu et al. (2017)
*R.grisea*	PC 2-1129IS75	Europe	AY061679	Miller and Buyck (2002)
*R.heterophylla*	PC 209RUF24	Europe	AY061681	Miller and Buyck (2002)
*R.hortensis*	IB 1997/0787	Italy	HG798528	–
*R.ilicis*	PC 563IC52	Europe	AY061682	Miller and Buyck (2002)
*R.illota*	MICH 73719	France	KF245509	–
	UE 26.07.2002-3	Sweden	DQ422024	Eberhardt (2002)
*R.inamoena*	107	Italy: Punta Chiappa	KJ834597	Melera et al. (2016)
	109	Italy: Punta Chiappa	KJ834595	Melera et al. (2016)
***R.indocatillus***	**HGAS-MF 009917**	**China: Guizhou**	**MN649191**	This study
	**HGAS-MF 009903**	**China: Guizhou**	**MN649192**	This study
*R.indocatillus*	AG 18-1653	India: Uttarakhand	MN581165	Ghosh et al. (2020)
*R.insignis*	HMAS 267740	China: Heilongjiang	KF850404	Li (2014)
	PC Buyck 00.2149	Europe	AY061700	Miller and Buyck (2002)
*R.mistiformis*	JC170305	Spain: Castilla-Leon	MK105677	Vidal et al. (2019)
	AMC H-69	Spain: Castilla-Leon	MK105680	Vidal et al. (2019)
*R.mutabilis*	BHI-F384a	USA: Massachusetts	MF161239	Haelewaters et al. (2018)
	DPL 10654	USA: Texas	KF810137	–
*R.neerimea*	MEL2101871	Australia: Victoria	EU019915	Lebel and Tonkin (2007)
*R.nondistincta*	OSC 62139	USA: Oregon	KP859276	–
*R.obscuricolor*	KD 16-30	India: Sikkim	MF805816	Das et al. (2017)
	KD 16-22	India: Sikkim	MF805817	Das et al. (2017)
*R.oleifera*	TU 116011	Benin	UDB016936	–
	TU 102082	Zambia	UDB013811	–
*R.ombrophila*	86	Spain	KF971694	Melera et al. (2016)
*R.parksii*	Trappe 14997	USA	AY239335	–
*R.pectinata*	PC Buyck 2304	Europe	AY061706	Miller and Buyck (2002)
	2010BT02	Germany	KF318081	Melera et al. (2016)
	2010BT48	Germany	KF318082	Melera et al. (2016)
*R.pectinatoides*	MICH 52692	USA: Tennessee	KF245518	–
	HMAS251202	China: Yunnan	JX425405	Li (2014)
	NYS2303.1	USA: New York	KU640189	Melera et al. (2016)
*R.pila*	MA-Fungi 30667	Spain	AF230893	Calonge and Martín (2000)
*R.pilosella*	BRI-H5974	Australia: Queensland	EU019941	Lebel and Tonkin (2007)
*R.praetervisa*	UE 2006-11-12-01	Italy	UDB019333	–
	IB 1997-0812	Italy	UDB019331	–
*R.pseudocatillus*	GDGM 75338	China: Guangdong	MK049974	Yuan et al. (2019)
	K 15060706	China: Guangdong	MK049975	Yuan et al. (2019)
*R.pseudopectinatoides*	HMAS 265020	China: Xizang	KM269079	Li et al. (2015b)
	HMAS 251523	China: Xizang	KM269077	Li et al. (2015b)
*R.pulverulenta*	PC BB2004-245	USA: Tennessee	EU598186	–
*R.punctipes*	K 17052318	China: Guangdong	MH168576	Yuan et al. (2019)
	K 16051001	China: Guangdong	MH168577	Yuan et al. (2019)
*R.putida*	IB 1997/0791	Italy	HG798527	–
*R.recondita*	UPS AT2001049	Sweden	DQ422026	Eberhardt (2002)
	WGS 84	Switzerland	KJ530750	Melera et al. (2016)
	TU106223	Estonia: Saare maakond	UDB011156	–
*R.rufobasalis*	H15060622	China: Guangdong	MH168567	Song et al. (2018)
	H17052204	China: Guangdong	MH168570	Song et al. (2018)
*R.senecis*	SFC 20110921-18	Korea: Socho-myeon	KX574698	Lee et al. (2017)
	CUH AM102	India: West Bengal	KP142981	Khatua et al. (2015)
*R.shafferi*	OSC 51046	USA: Washington	AY239327	–
*R.similaris*	OSC 44426	USA: California	AY239329	–
	Trappe 7753	USA: Oregon	AY239349	–
*Russula* sp.	LHJ170913-01	China: Guangdong	MK860691	Song et al. (2020)
***R.straminella***	**HGAS-MF 009920**	**China: Guizhou**	**MN649194**	This study
	**HGAS-MF 009922**	**China: Guizhou**	**MN649195**	This study
	**HGAS-MF 009925**	**China: Guizhou**	**MN649189**	This study
*R.subfoetens*	HMJAU38006	China: Heilongjiang	KY681430	Liu et al. (2017)
	TU101908	Finland: Nilsiä	UDB016206	–
*R.subfulva*	Trappe 14998	USA: Oregon	AY239321	–
***R.subpectinatoides***	**HBAU15023**	**China: Jiangsu**	**MW041163**	This study
	**HBAU15024**	**China: Jiangsu**	**MW041164**	This study
	**HBAU15025**	**China: Jiangsu**	**MW041165**	This study
	**HBAU15026**	**China: Jiangsu**	**MW041166**	This study
*R.subpunctipes*	RITF 2616	China: Guangdong	MK860692	Song et al. (2020)
	RITF 2617	China: Guangdong	MK860693	Song et al. (2020)
*R.substriata*	HKAS 102278	China: Yunnan	MH724921	Wang et al. (2019)
***R.succinea***	**HGAS-MF 009909**	**China: Guizhou**	**MN649196**	This study
	**HGAS-MF 009904**	**China: Guizhou**	**MN649188**	This study
	**HGAS-MF 009906**	**China: Guizhou**	**MN649198**	This study
	**HGAS-MF 009915**	**China: Guizhou**	**MN649190**	This study
*R.succinea*	HFJAU0301	China: Jiangxi	MN258682	–
*R.ventricosipes*	PC 0142480	USA	KY800364	Buyck et al. (2017)
*R.vinaceocuticulata*	PDD 64246	New Zealand	GU222258	–

Note: Species, specimens and GenBank accession numbers in bold are newly collected and sequenced in this study.

## Results

### Phylogenetic analyses

A total of 112 ITS sequences (107 of sect. Ingratae and 5 of out-groups), including 13 newly-generated ones, were analysed in this study. The alignment for ITS phylogenetic analyses was composed of 543 characters including gaps. Of these characters in the matrix, 266 were variable, 201 were parsimony-informative, 65 variable characters were parsimony-uninformative. The parameters of MP analysis were CI 0.444, HI 0.784, RI 0.784, RC 0.348 and TL 869. The most suitable model for BI and MP analyses is GTR+I+G.

The resulting MP, ML and BI phylograms are consistent in topology of highly supported basal ranks (Clades A, B, C, D, E, F, H and I); thus, only the MP tree is presented in Fig. 1. A total of nine complexes and 24 species rank clades can be recognised with high support values. The 11 Chinese sequences were grouped in three clades that were further described as new species of *R.straminella*, *R.subpectinatoides* and *R.succinea*. High bootstraps and posterior probabilities supporting these clades are distinctly independent from those of other known taxa. Clades H, F and C in Fig. 1 generally corresponded with clades 1, 4 and 3 of Lee et al. (2017), in which species in Clade 2 are represented by Clades E and I in this study. The Indian and Chinese specimens of *R.indocatillus* clustered together and formed a strongly supported, distinct clade (MLBS 96, PP 0.99, MPBS 89). The new species, *R.straminella*, formed an independent lineage in Clade F. The concrete phylogenetic status of *R.straminella* still remains unsolved in ITS sequence analyses. The new species *R.succinea* and two North American specimens were identified as *R.foetentula* with passable support (MPBS 58, PP 1). The new species, *R.subpectinatoides*, clustered with a majority of members from clade C and formed a highly supported clade (MLBS 96, PP 1, MPBS 88). The close relationship with *R.pseudopectinatoides* indicated in similarity searching was not supported in phylogenetic topologies.

The DNA sequence similarity search results for the ITS1–5.8S-ITS2 region of the new species are as follows: two North American specimens of gasteroid *R.similaris* Trappe & T.F. Elliott (AY239349 and KC152107) had the highest sequence identity (98.2%) to the new species *R.straminella*, then *R.nondistincta* Trappe & Castellano (KP859276) (98.1%); *R.pseudopectinatoides* (KM269079) had the highest sequence similarity (98%) to the new species *R.subpectinatoides*, then *R.praetervisa* Sarnari (95%); *R.foetentula* Peck (KJ834623) had the highest sequence identity (96.9%) to the new species *R.succinea*, then *R.subfoetens* W.G. Sm. (UDB016206) (94%). The Chinese collections of *R.indocatillus* had sequence identities of 99% to its type specimens (MN581483 and MN581165) from India.

### Taxonomy

#### Russula indocatillus

Taxon classificationFungi RussulalesRussulaceae

A. Ghosh, K. Das & R.P. Bhatt, Nova Hedwigia 111(1–2): 124. 2020.

98CB8498-1A76-5000-96D8-1103B809C8A0

**Figure 2.**
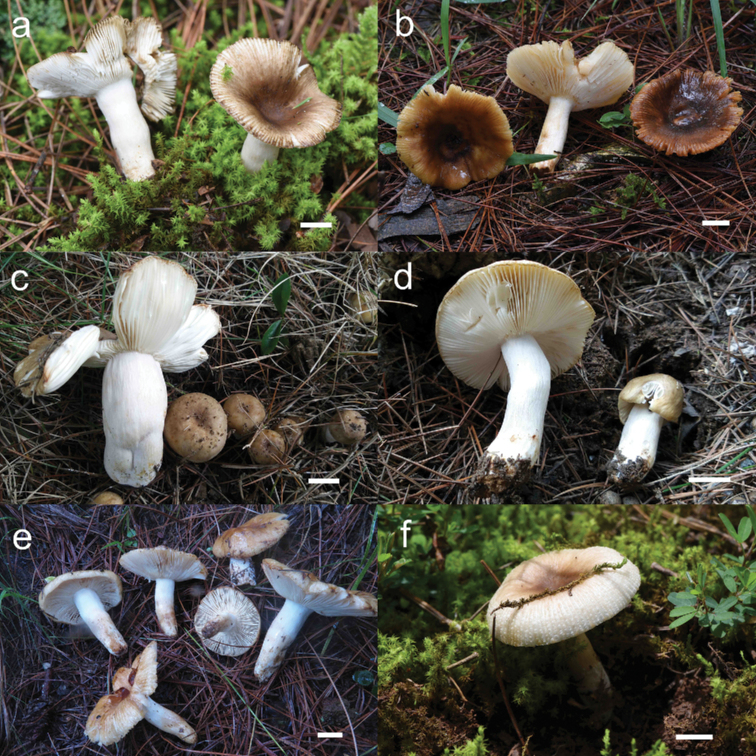
Basidiomata **A***Russulaindocatillus***B***R.straminella***C–D***R.subpectinatoides***E–F***R.succinea*. Bars: 10 mm.

**Figure 3.**
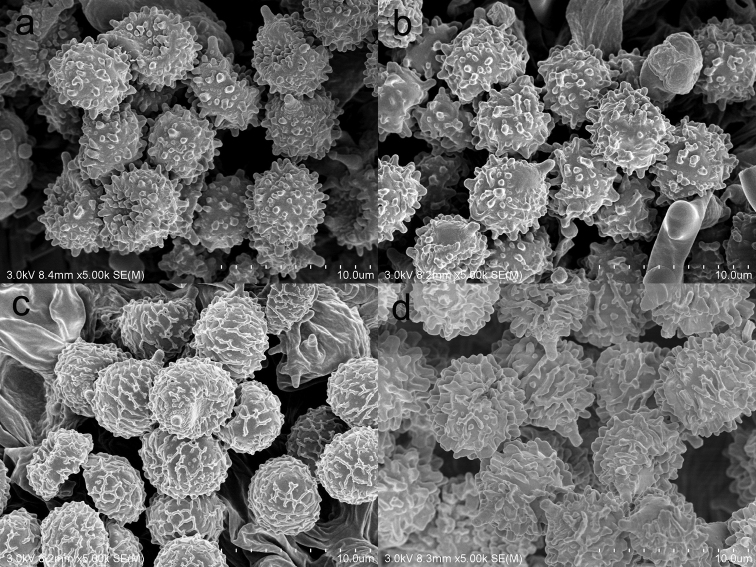
SEM photo of basidiospores **A***Russulaindocatillus***B***R.straminella***C***R.subpectinatoides***D***R.succinea*.

**Figure 4.**
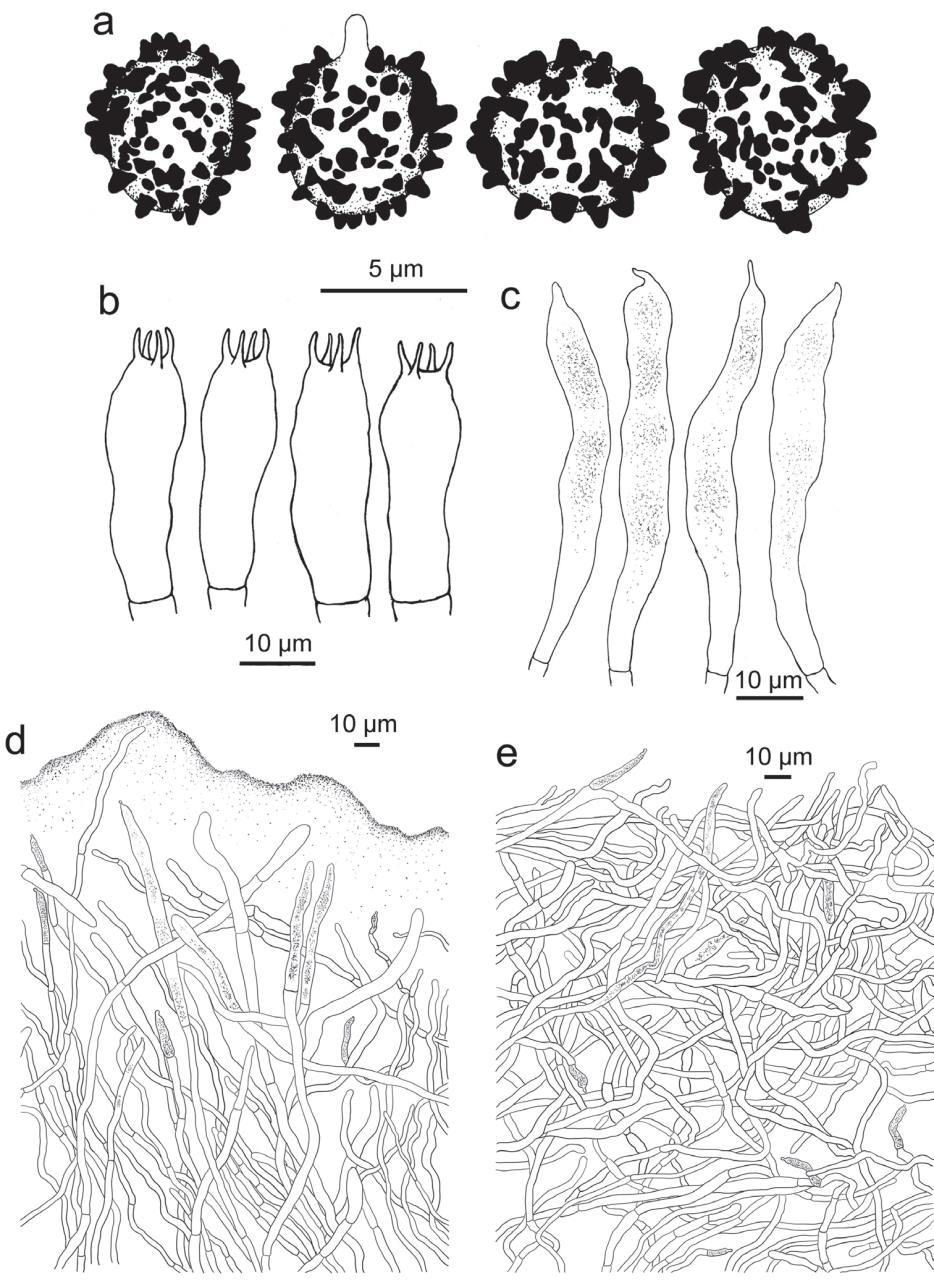
*Russulaindocatillus*, holotype **A** basidiospores **B** basidia **C** hymenial cystidia**D** suprapellis in pileus centre **E** suprapellis in pileus margin.

**Figure 5.**
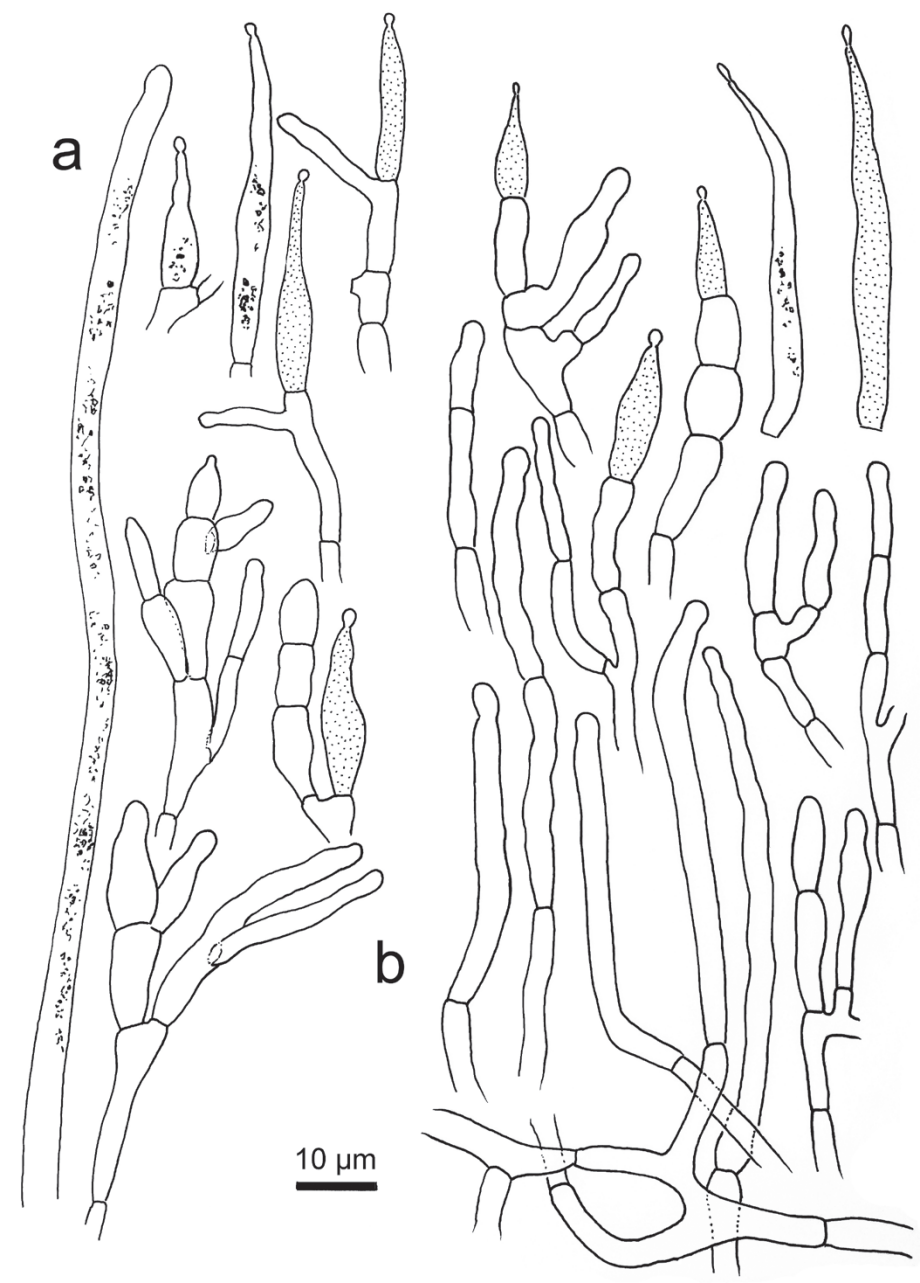
*Russulaindocatillus*, holotype **A** hyphal extremities in pileipellis margin **B** hyphal extremities in pileus centre.

Figs 2a , 3a , 4 and 5.

*Basidiomata* small to medium sized. Pileus 35–46 mm in diam., hemispherical when young, then plano-convex to applanate, depressed at centre when mature, rarely infundibuliform, viscid when wet, brownish tinged, intermixed with greyish-yellow fringe, Verona Brown (XXIX13′′k), Chocolate (XXVIII7′′m), to Cinnamon Brown (XV15′k) at centre, sometimes with a tinge of Argus Brown (III13m) or Brussels Brown (III15m), Pecan Brown (XXVIII13′′i) or Hazel (XIV11′′k) when old and dry; margin acute, slightly incurved first, straight when mature, slightly undulate, often cracked, tuberculate-striate 10–15 mm from the edge inwards, peeling 1/5–1/4 towards the centre, Ochraceous Tawny (XV15′i), Mikado Brown (XXIX13′′i) or Tawny Olive (XXIX17′′i) when young, often Avellaneous (XL17′′′b), Cinnamon (XXXI15′′) to Clay Colour (XXIX17′′) when mature. *Lamellae* adnate, rarely sub-free, 2–5 mm in height at mid-radius of pileus, fragile, rarely forked near the stipe, interveined, pale cream tinged, White (LIII) when young, Cream Colour (XVI19′f) in age, often stained yellowish to brownish with Buckthorn Brown (XV17′i) to Yellow Ochre (XV17′); edge even, narrowing towards the pileus margin, 9–16 per cm near the pileus margin; lamellulae rare. *Stipe* subcentral to central, 2.5–4.7 × 1–1.4 cm, cylindrical to subclavate, rarely tapered towards the base, annulus absent, first smooth, then often longitudinally rugulose in age, White (LIII), rarely stained with brownish tinge of Aniline Yellow (IV19i) to Honey Yellow (XXX19′′), first stuffed, hollow when mature. *Context* 2–4 mm thick at the centre of pileus, initially White (LIII), Light Ochraceous-Salmon (XV13′d) to Primuline Yellow (XVI19′) when mature, unchanging or slowly turning Ochraceous-Tawny (XV15′) to Buckthorn Brown (XV17′i) when injured or touched, brittle; taste mild, rarely slightly acrid when young; odour indistinct. *Spore print* cream-coloured (Romagnesi IIc–IId).

*Basidiospores* [200/8/4] (4.9–) 5.3–6.8 (–7.3) × (4.7–) 5.0–5.9 (–6.3) μm, Q = (1.01–) 1.05–1.28 (–1.33) (**Q** = 1.18 ± 0.08), 6.1 × 5.5 μm in average, subglobose to broad ellipsoid, ornamentation composed of conical to verrucous amyloid warts of very different sizes, mostly isolated, rarely linked as short ridges or with occasional line connections, not reticulate, warts 0.7–1 μm in height; suprahilar spot inamyloid and indistinct. Basidia 27–39 × 8–9 μm, hyaline in KOH, clavate to subclavate, four-spored, projecting 15–20 μm beyond the hymenium; sterigmata 3–6 μm, pointed, often straight, slightly tortuous towards the tip. Hymenialcystidia rare, less than 200/mm^2^, 56–70 × 6–9 μm, fusiform to subclavate, rarely subcylindrical, thin-walled, projecting 20–40 μm beyond the hymenium, apex often mucronate, contents sparse, unevenly distributed, granular, greyish in SV. Pileipellis two layered, composed of suprapellis (80–150 μm thick) and subpellis (100–150 μm thick). Suprapellis an ixotrichoderm at pileus centre, composed of oblique to erect, septate, hyaline hyphae; acid-resistant encrustations absent, terminal cells cylindrical to subcylindrical, apex obtuse, rarely mucronate, mostly 40–70 μm in length; pileus margin a trichoderm composed of repent to tilted elements, terminal cells mostly 7–20 (–25) μm in length, ampullaceous, ellipsoid or cylindrical, obtuse to mucronate at apex, longer terminal cells similar to those in pileus centre also present; subapical cells contain islands of more or less inflated, 2–4 septate cells. Pileocystidia present in suprapellis and subpellis, abundant at pileus centre, dispersed at margin, one-celled, subulate, lageniform, fusiform, cylindrical, rarely appendiculate, 4–6 μm in width, many in suprapellis 15–25 μm in length, others up to 60 μm, even reaching a length of 100 μm in subpellis, apex mucronate, acicular to lanceolate in suprapellis, obtuse in subpellis, contents granulate, sparse, greyish in SV. Subpellis composed of repent to irregularly interlaced, inflated, septate hyphae 3–5 μm wide. Clamp connections absent in all tissues.

##### Specimens examined.

China, Guizhou Province, Weining Yi, Hui, and Miao Autonomous County, Caohai National Nature Reserve, 26°53'N, 104°12'E, alt. 2171 m, on the ground in coniferous forest, 9 September 2017, C.Y. Deng A (HGAS-MF 009903); ibid, alt. 1987 m, C.Y. Deng dcy2306 (HGAS-MF 009918); ibid, alt. 2053 m, C.Y. Deng dcy2303 (HGAS-MF 009911); ibid, alt. 2106 m, C.Y. Deng CH2017090971 (HGAS-MF 009917).

##### Habit and habitat.

Single to scattered on yellow brown soil in coniferous forest dominated by *Pinusarmandii* and *P.yunnanensis* at 1900–2200 m altitude.

##### Distribution.

China (Guizhou) and India (Uttarakhand).

##### Notes.

The Chinese collections fit well with the original description of Ghosh et al. (2020), except for a few differences. The Indian specimens have longer basidia, 35–60 × 9–11 μm. The original description of *R.indocatillus* also noted that the type specimen was collected in a temperate mixed forest with *Myrica*, *Quercus* and *Rhododendron*. The coniferous tree species in this habitat were not mentioned. The Chinese collection is from a subalpine coniferous forest of subtropical region dominated by *Pinus* spp. with the main undergrowth species of *Berberiscavaleriei*, *Corylusyunnanensis*, *Elaeagnusumbellata* and *Rosa sweginzowii* (He et al. 2019).

Amongst the closely-related species in Clade H, *R.amoenolens* Romagn. and *R.cerolens* Shaffer have a strongly acrid taste, disagreeable sub-spermatic odour, basidiospore length up to 9 μm and longer hymenial cystidia up to 100 μm (Shaffer 1972; Romagnesi 1985; Sarnari 1998); *R.catillus* lacks lamellulae, has longer basidia 42–49 × 9.3–11.7 μm, shorter pileipellis terminal cells 41–72 × 3–7 μm and lacks pileocystidia (Lee et al. 2017); *R.pseudocatillus* has larger basidiospores 7–9.2 × 5.1–6.7 μm with higher ornamentation (up to 1.2 μm) which is never reticulate (Yuan et al. 2019).

Some members of R.sect. Ingratae, which were originally described from Himalayan Mountains and adjacent south-western China, may be confused with *R.indocatillus* in the field. Their main morphological differences are as follows: *R.abbotensis* has a crustose to areolate pileus with purplish-red to reddish-brown tinges, an ixotrichoderm pileipellis with pileocystidia 5 μm in width and an occurring in ectomycorrhizal association with *Quercus* spp. (Das and Sharma 2005); *R.arunii* can be distinguished by its fishy odour, amyloid suprahilar spot, 3–4 μm wide pileocystidia, mostly with a capitate apex and habitat in a tropical rain forest of *Pterygotaalata* (Crous et al. 2017); *R.ahmadii* has larger basidiospores (5.6–) 6.1–9.2 (–9.4) × (5–) 5.1–6 (–6.5) μm with low (up to 0.3 μm high), partly reticulated ornamentation and cutis type of pileipellis (Jabeen et al. 2017); *R.foetentoides* can be distinguished from *R.indocatillus* by its smooth pileus margin, absence of lamellulae and its basidiospore ornamentation of 1.7–2 µm in height (Razaq et al. 2014); *R.natarajanii* differs in having larger basidiospores, 6.8–8.8 × 5.8–7.1 μm and longer hymenial cystidia, 60–90 × 6–10.5 μm (Das et al. 2006); *R.pseudopectinatoides* has larger basidiospores (6–) 6.5–9 (–9.5) × (5–) 5.1–6 (–6.5) μm with partly reticulate ornamentation, longer hymenial cystidia up to 90 μm and terminal cells of suprapellis hyphae often with obtuse to ventricose apex (Li et al. 2015b); *R.succinea* differs in larger basidiospores with incompletely reticulated ornamentations, longer basidia and pileocystidia up to 10 μm in width (Figs 10 and 11); *R.tsokae* can be distinguished from *R.indocatillus* by its larger basidiomata 8–13 cm in diam., yellowish-orange tinged stipe and larger basidiospores 6.8–8.8 × 5.8–7.1 μm with reticulated ornamentation up to 2 μm high (Das et al. 2010).

#### Russula straminella

Taxon classificationFungiRussulalesRussulaceae

G.J. Li & C.Y. Deng sp. nov.

E18E5240-9B90-5A19-9A9C-9E79AB789F26

Fungal Names: FN 570758

**Figure 6.**
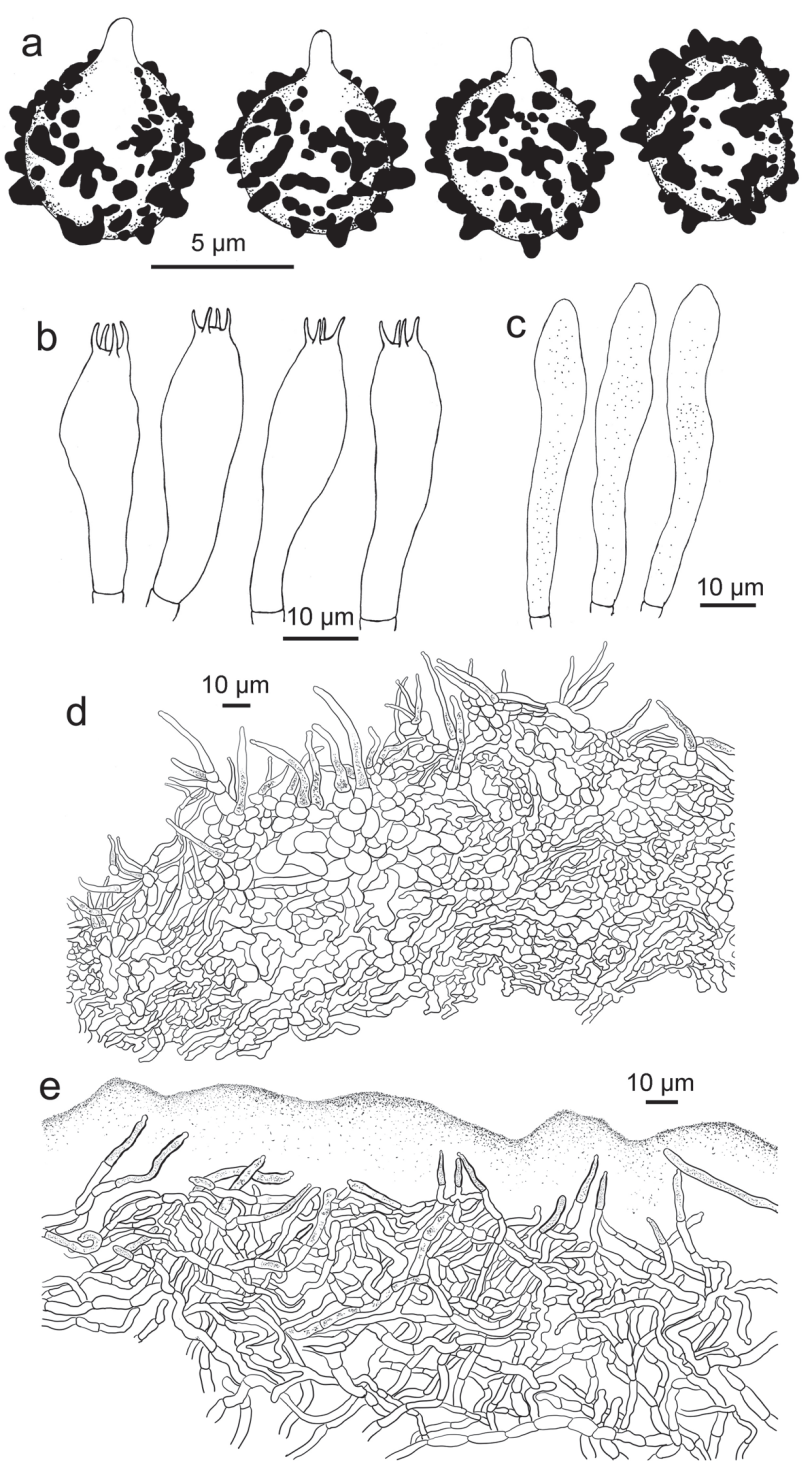
*Russulastraminella*, holotype **A** basidiospores **B** basidia **C** hymenial cystidia**D** suprapellis and partial subpellis in pileus centre **E** suprapellis in pileus margin.

**Figure 7.**
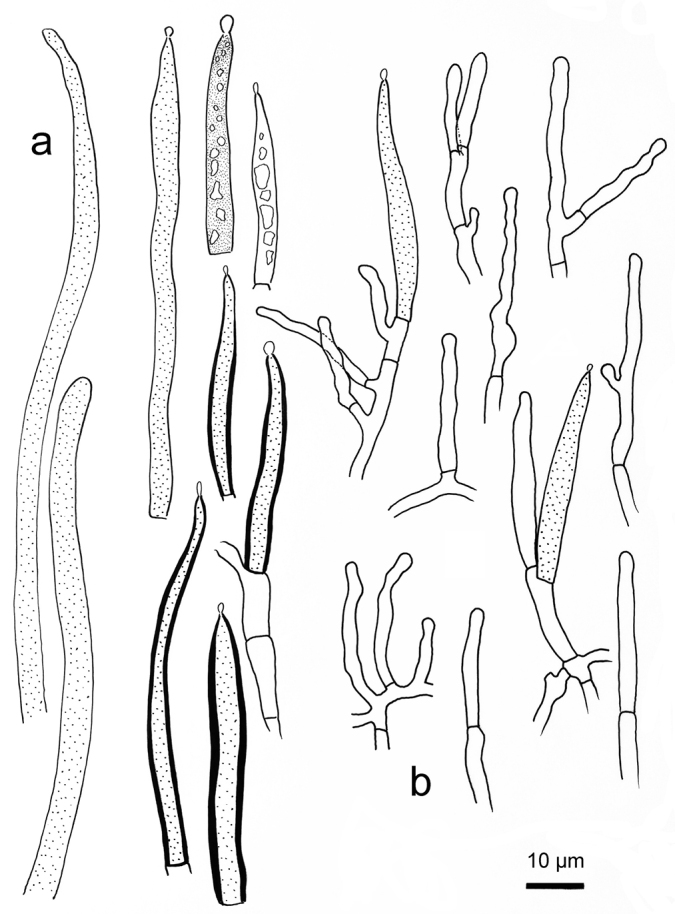
*Russulastraminella*, holotype **A** hyphal ends in pileipellis margin **B** hyphal ends in pileus centre.

Figs 2b , 3b , 6 and 7.

##### Etymology.

referring to the yellowish tinged pileus

##### Holotype.

China, Guizhou Province, Guiyang City, Yunyan District, Guizhou Botany Garden, 26°37'N, 106°43'E, alt. 1107 m, on the ground in coniferous forest, 8 July 2017, C.Y. Deng 2017–209 (HGAS-MF 009922, **Holotype**). GenBank accession: MN649195 (ITS).

##### Diagnosis.

This species is characterized by the yellow, brownish-yellow to brown pileus, tuberculate-striate margin, adnate lamellae tinged ochraceous when bruised, rare lamellulae, white stipe turning brownish-yellow when injured, mild to rarely acrid context, cream spore print, globose, subglobose to broad ellipsoid basidiospores (5.4–) 5.8–7.1 (–7.6) × (4.7–) 5.1–6.5 μm, 6.4 × 5.6 μm on average, with verrucous to conical, partly reticulate ornamentations 0.7–1 μm in height, subclavate to clavate basidia 33–40 × 9–11 μm, clavate to subclavate hymenial cystidia 56–70 × 8–10 μm, a suprapellis composed of two layers, a trichoepithelium at pileus centre and an ixotrichoderm towards the margin, pileocystidia abundant at pileus centre, but sparse in margin, a cutis type of subpellis and habitat on the ground in coniferous forests.

##### Description.

*Basidiomata* small to medium sized. Pileus 33–57 mm in diam., initially flat to hemispherical, then plano-convex to applanate, finally often concave at centre, gelatinised, yellowish to brownish-yellow tinged, intermixed with brownish fringe, Argus Brown (III13m), Warm Sepia (XXIX13′′m), to Verona Brown (XXIX13′′k) at centre, rarely with a paler tinge of Mikado Brown (XXIX13′′i), Rood’s Brown (XXVIII11′′k) to Cacao Brown (XXVIII9′′i); margin acute to subacute, enrolled when young, often undulate, sometimes cracked when mature, tuberculate-striate 8–15 mm from the edge inwards, peeling 1/5–1/4 towards the centre, first Aniline Yellow (IV19i), Sayal Brown (XXIX15′′) to Cinnamon Buff (XXIX15′′d), finally Mikado Brown (XXIX13′′i), Snuff Brown (XXIX15′′k) to Clay Colour (XXIX17′′). *Lamellae* adnate, fragile, occasionally forked near the stipe and pileus margin, interveined, first White (LIII), then of Cream Colour (XVI19′f) when mature, often having an ochraceous tinge of Olive Ochre (XXX21′′), Isabella Colour (XXX19′′i) to Honey Yellow (XXX19′′) when bruised, taste mild to slightly acrid; edge even, narrowing towards the pileus edge, 8–16 pieces per cm in the edge; lamellulae rare. *Stipe* central, 3.5–6.5 × 1–1.5 cm, cylindrical, slightly tapering towards the base, annulus absent, first smooth, slightly longitudinally rugulose when mature, White (LIII) when young, turning a pale brownish-yellow tinge of Kaiser Brown (XIV9′k), Aniline Yellow (IV19i) to Buckthorn Brown (XV17′i) after bruising, initially stuffed, fistulous to hollow when mature. *Context* White first, slowly turning a pale ochraceous tinged of Yellow Ochre (XV17′) to Ochraceous-Buff (XV15′b) when injured, 2–4 mm thick at the centre of pileus, compact; taste mild, rarely slightly acrid, with no distinct odour. *Spore print* cream coloured (Romagnesi IIc–IId).

*Basidiospores* [150/6/3] (5.4–) 5.8–7.1 (–7.6) × (4.7–) 5.1–6.5 μm, Q = (1.00–) 1.03–1.28 (–1.31) (**Q** = 1.15 ± 0.07), 6.4 × 5.6 μm in average, globose, subglobose to broadly ellipsoid, rarely ellipsoid, ornamentation amyloid, composed of verrucous to conical warts 0.7–1 μm in height, often linked by fine lines as short ridges, partly reticulate, rarely isolated; suprahilar area inamyloid, but distinct. *Basidia* 33–40 × 9–11 μm, hyaline, often yellowish in KOH, subclavate to clavate, sometimes cylindrical, mostly with four sterigmata 4–7 μm long. *Hymenialcystidia* rare, less than 500/mm^2^, 56–70 × 8–10 μm, clavate to subclavate, rarely subfusiform, projecting 20–40 μm beyond hymenium, apex rounded, contents sparse, granular, evenly distributed, pale greyish in SV. *Pileipellis* two-layered, clearly distinguished from the subjacent sphaerocytes. *Suprapellis* 70–130 μm thick, acid-resistant encrustations absent, a trichoepithelium at pileus centre, partly an ixo-trichoepithelium, composed of erect to suberect hyphae, terminal cells cylindrical, 20–40 × 3–5 μm, obtuse at apex, partly ventricose, subapical cells sometimes inflated, rarely branched, 15–25 × 8–12 μm, an ixotrichoderm at pileus margin, composed of erect to ascending, rarely repent hyphae, terminal cells 30–55 × 3–5 μm, cylindrical, often thick-walled, tapered to mucronate at apex. *Pileocystidia* abundant, often fasciculate at pileus centre, narrowly lanceolate to bayonet-shaped, 30–60 × 5–8 μm, one-celled, contents granular, blackish-grey in SV. Pileocystidia sparse at the pileus margin, cylindrical, 4–8 μm in width, slightly tapered at apex, contents grey in SV. *Subpellis* composed of loosely interwoven, mostly repent, septate hyphae often inflated, 3–8 μm in width. *Clamp connections* absent in all tissues.

##### Additional specimens examined.

China, Guizhou Province, Guiyang City, Yunyan District, Guizhou Botany Garden, 26°37'N, 106°43'E, alt. 1074 m, on the ground in coniferous forest, 8 July 2017, C.Y. Deng dcy2305 (HGAS-MF 009920, paratype); ibid, alt. 1385 m, C.Y. Deng dcy2302 (HGAS-MF 009925, paratype).

##### Habit and habitat.

Single to scattered on yellow brown soil in coniferous forest dominated by *Pinusarmandii* and *P.massoniana* at 1100–1400 m altitude.

##### Distribution.

China (Guizhou).

##### Notes.

This new species can be distinguished from members of R. sect. Ingratae described from China and the Himalayan region as follows: *Russulagelatinosa*, *R.guangdongensis* Z.S. Bi & T.H. Li, *R.punctipes*, *R.senecis*, *R.subpunctipes* and *R.tsokae* have basidiospore ornamentation composed of high wings (often above 2 μm), ranging over long distances or even encircling (Bi and Li 1986; Song et al. 2018, 2020). The Asian species of R.sect. Ingratae, *R.ahmadii*, *R.natarajanii* and *R.pseudopectinatoides* have basidiospore ornamentation lower than 0.7 μm (Das et al. 2006; Li et al. 2015b; Jabeen et al. 2017). For species that have similar basidiospore ornamentation, *R.abbotensis* has reddish-brown to purplish-red tinges on pileus surface, pruinose to scurfy stipe at base, larger basdiospores, 8–10 × 7.3–8.5 μm and hymenial cystidia with mucronate apices (Das and Sharma 2005); *R.arunii* has pileus turning light orange to greyish-orange when old, context having a fishy odour and narrow pileocystidia 3–4 μm in width (Crous et al. 2017); *R.indocatillus* has hymenial cystidia with mucronate, capitate, moniliform, rostrate or appendiculate apex with cylindrical or slightly inflated subapical cells (Ghosh et al. 2020); *R.obscuricolor* has a pale yellowish-white tinge in pileus margin, pungent and bitterish context, narrow pileocystidia 3–5 μm in width (Das et al. 2017); *R.pseudocatillus* has greyish-brown pileus centre, towards the margin very pale yellow, larger basidiospores, 7–9 μm in diam. and narrower pileocystidia (3–6 μm in width) unchanging in SV (Yuan et al. 2019); *R.rufobasalis* has reddish stipe base, mucronate or appendiculate apex of hymenial cystidia and thick-walled terminal cells (Song et al. 2018).

#### Russula subpectinatoides

Taxon classificationFungiRussulalesRussulaceae

G.J. Li & Q.B. Sun sp. nov.

059F42CB-ED1F-5956-8D40-7AE126C9407A

Fungal Names: FN 570759

**Figure 8.**
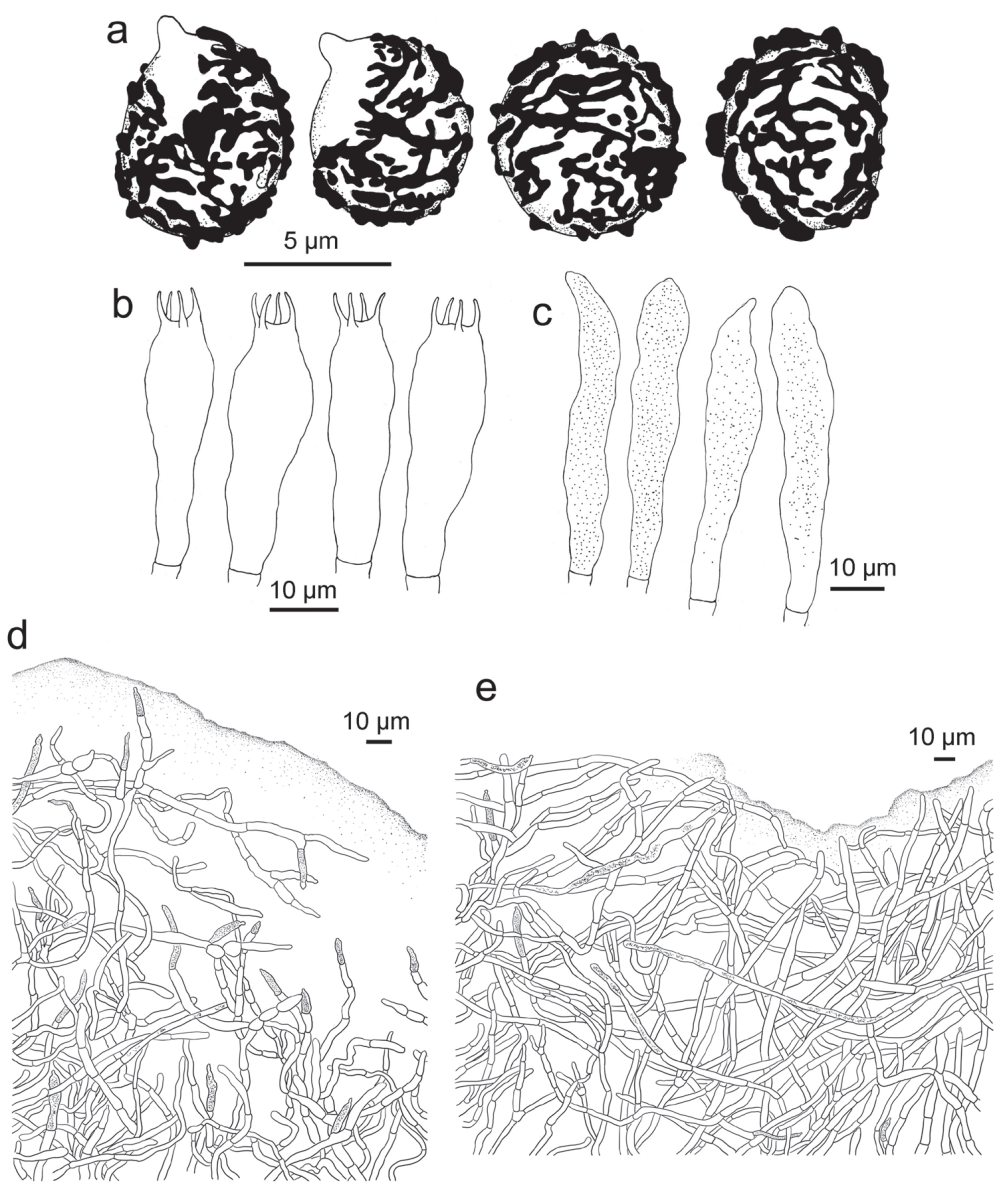
*Russulasubpectinatoides*, holotype **A** basidiospores **B** basidia **C** hymenial cystidia**D** suprapellis in pileus centre **E** suprapellis in pileus margin.

**Figure 9.**
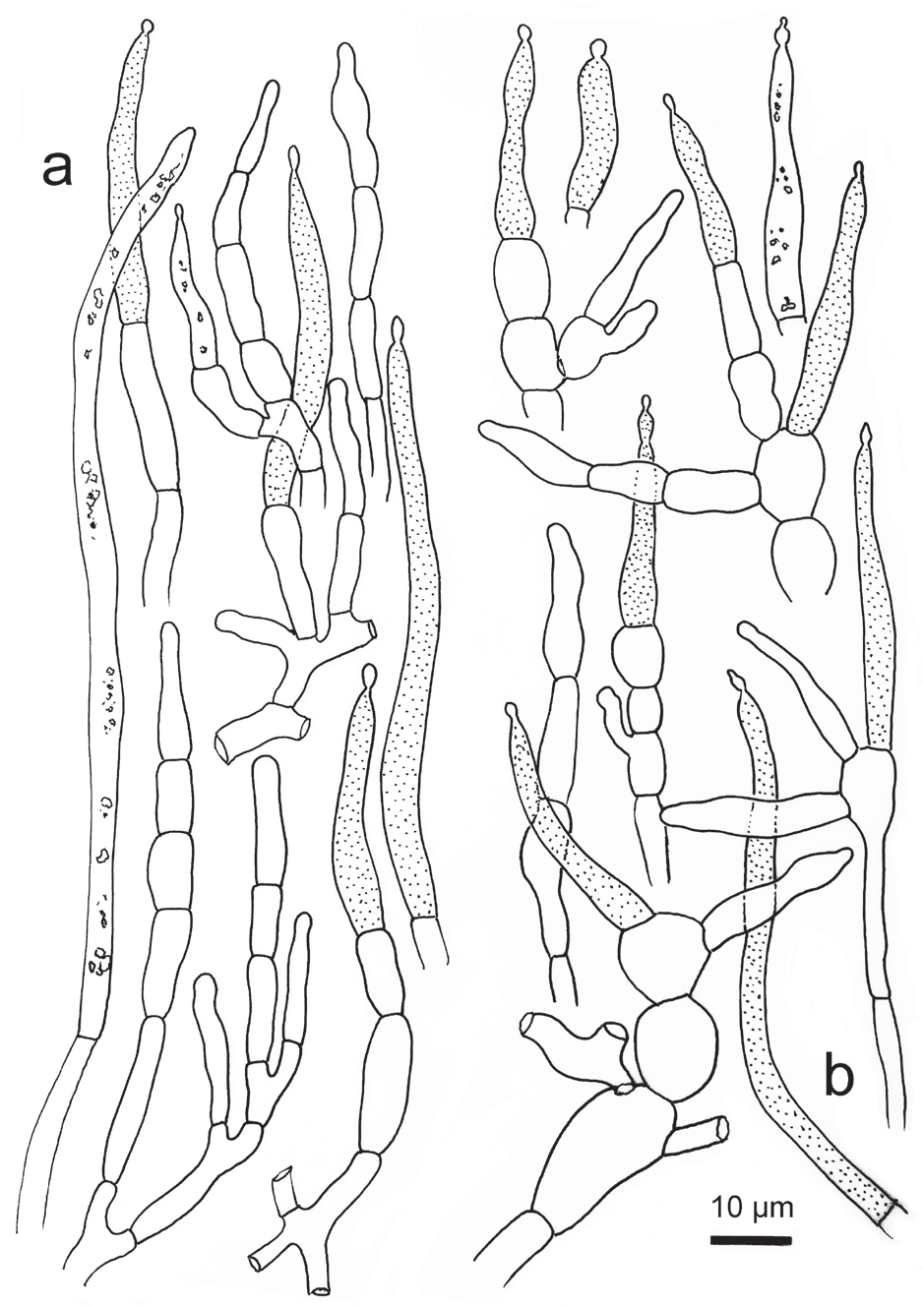
*Russulasubpectinatoides*, holotype **A** hyphal extremities in pileipellis margin **B** hyphal extremities in pileus centre.

Figs 2c , 2d , 3c , 8 and 9.

##### Etymology.

named for its morphological resemblance to *R.pectinatoides* Peck.

##### Holotype.

China, Jiangsu Province, Nanjing City, Qixia District, Nanjing Normal University, 32°06'N, 118°54'E, alt. 84 m, on the ground in coniferous forest, 28 August 2018, Q.B. Sun 2018001 (HBAU15030, **Holotype**). GenBank accession: MW1041163 (ITS).

##### Diagnosis.

This species is characterised by the greyish-brown to brownish-yellow pileus, striate margin, adnate to subadnate lamellae rarely staining reddish-brown when bruised, infrequent lamellulae, context slowly turning pale ochre after injury and slightly to moderately acrid taste, cream spore print, subglobose to broadly ellipsoid basidiospores (5.3–) 5.6–6.3–7 (–7.3) × (4.1–) 4.6–5.2–6 (–6.3) μm, ornamentation 0.3–0.5 μm in height, composed of long ridges forming an incomplete to complete reticulum, fusiform to subclavate, basidia 27–50 × 8–12 μm, fusiform to subclavate hymenial cystidia 56–73 × 6–12 μm, pileipellis with one-celled, slender, mucronate, conical, needle-shaped to cylindrical pileocystidia, 5–7 μm in width; and habitat in coniferous forest.

##### Description.

*Basidiomata* small to medium-sized. *Pileus* 18–95 mm in diam., initially hemispherical, concave at centre, turning applanate with age, often depressed at stipe, slightly viscous when young or humid, greyish-brown to brownish-yellow tinged, intermixed with dark brown fringe, Buffy Citrine (XVI19′k) to Light Brownish Olive (XXX19′′k) at centre, Citrine-Drab (XL19′′′i), Drab (XLVI17′′′′) to Benzo Brown (XLVI13′′′′i) when mature, often turning Buffy Olive (XXX21′′k) to Saccardo’s Olive (XVI19′m) when old and dry; margin acute to subacute, involute when young, straight with maturity, sometimes dehiscent, undulate to curled-up when old, striate 1/4–1/3 towards the centre, not or rarely weakly tuberculate, peeling 1/5–1/3 towards the centre, rarely flaking in small patches, with an ochre tinge of Old Gold (XVI19′i), Olive Ochre (XXX21′′) to Tawny-Olive (XXIX17′′i). *Lamellae* adnate to subadnate, 3–6 mm in height at the midpoint, sometimes forked near the stipe and the pileus edge, interveined, white to pale cream, White (LIII) when young, Light Buff (XV17′f) to Cream Colour (XVI19′f) with age, rarely stained reddish-brown tinge of Buckthorn Brown (XV17′i) when bruised, taste slightly to moderately acrid; edge even, constricted towards the margin, 9–19 pieces per cm at the edge; lamellulae infrequent. *Stipe* central to subcentral, 2.4–9.3 × 1.3–2.7 cm, slightly narrowing towards the base and apex, smooth at first, longitudinally slightly rugulose when mature, White (LIII) first, sometimes faintly stained with Honey Yellow (XXX19′′) to Olive Ochre (XXX21′′) when bruised, stuffed first, fistulous to hollow when old. Context 2–5 mm thick above the stipe, initially White (LIII), unchanging or slowly turning pale ochre tinge of Cinnamon Buff (XXIX15′′d) when bruised, pale greyish-yellow tinge of Cartridge Buff (XXX19′′f) at base when old, taste slightly to moderately acrid, with no distinct odour. Spore print cream coloured (Romagnesi IIc–IId).

*Basidiospores* [250/10/5] (5.3–) 5.6–7 (–7.3) × (4.1–) 4.6–6 (–6.3) μm, Q = (1.02–) 1.05–1.31 (–1.37) (**Q** = 1.19 ± 0.09), 6.3 × 5.2 μm in average, mostly subglobose to broadly ellipsoid, rarely globose and ellipsoid, ornamentation amyloid, composed of long ridges forming an incomplete to complete reticulum, rarely intermixed with an isolated conical to verrucous warts and short crests, 0.3–0.5 μm in height; suprahilar spot inamyloid and indistinct. *Basidia* 27–50 × 8–12 μm, hyaline in KOH, subcylindrical to subclavate, rarely clavate or subfusiform, inflated towards the upper end or mid-piece, 4-spored, projecting 15–30 μm beyond hymenium; sterigmata 3–6 μm, slightly tortuous, sometimes straight. *Hymenialcystidia* sparsely distributed, fewer than 200/mm^2^, 56–73 × 6–12 μm, fusiform to subclavate, projecting 20–40 μm beyond the hymenium, contents granular, sparsely distributed, slightly greyish in SV; apex subacute, rarely obtuse; lamellar edge sterile. *Pileipellis* two layered, composed of suprapellis (80–140 μm thick) and subpellis (100–150 μm thick). Suprapellis an ixotrichoderm, composed of gelatinised, ascending to vertical, septate hyphae, acid-resistant encrustations absent, terminal cells mostly lanceolate to bayonet-shaped at pileus centre, mostly tapered at apex, rarely cylindrical, 20–30 × 4–7 μm, subapical cells sometimes inflated, barrel-shaped, ellipsoid or almost subglobose to globose; when compared with suprapellis at pileus centre, its margin is also an ixotrichoderm, but contains more repent elements, 3–5 μm in width, inflated hyphal cells not observed, lateral short ramifications frequent; pileocystidia long, cylindrical, non-septate, 3–5 μm in width, apex mucronate, contents granulate, sparse, pale grey in SV. Subpellis*a* composed of cylindrical, sometimes inflated, septate, loosely intricate, gelatinous, hyaline hyphae 3–6 μm in width. *Clamp connections* absent in all parts.

##### Additional specimens examined.

China, Jiangsu Province, Nanjing City, Qixia District, Nanjing Normal University, 32°06'N, 118°54'E, alt. 84 m, on the ground in coniferous forest, 28 August 2018, Q.B. Sun 2018002 (HBAU15031, paratype); ibid, 2018003 (HBAU15032, paratype); ibid, 2018004 (HBAU15033, paratype).

##### Habit and habitat.

Single to scattered on yellow brown soil in coniferous forest of subtropical monsoon climate zone dominated by *Cedrusdeodara*, *Pinusparviflora* and *P.thunbergii*.

##### Distribution.

China (Jiangsu).

##### Notes.

This new species is similar to *R.pseudopectinatoides* in its brownish-yellow pileus, slightly acrid taste, cream spore print, spores with low, subreticulate ornamentation and gelatinous pileipellis. It is notable that basidiomata of *R.subpectinatoides* were collected from a forest of introduced coniferous tree species. *Cedrusdeodara* is native in the western Himalayas, while *Pinusparviflora* and *P.thunbergii* are naturally distributed in the Japanese archipelago and Korean peninsula. Therefore, this new taxon may also occur in these introduced areas with its accompanying trees.

The Asian species of sect. Ingratae already recognizable by their long slender stipe, such as *R.gelatinosa*, *R.guangdongensis*, *R.punctipes*, *R.senecis*, *R.subpunctipes* and *R.tsokae* and cannot be confused with our new species, even more so because they have basidiospores composed of long wings, 2 μm high or more (Song et al. 2018, 2020). A similarly-winged spore ornamentation also differentiates species of the *R.grata* lineage which, moreover, usually have a distinct bitter almond smell. The more golden yellow pileus of species in the *R.foetens* or *R.subfoetens* lineages also avoids confusion with our new species and because many of these are distinctly very acrid. The strong yellowish stipe base that turns immediately red with KOH easily allows one to distinguish the few species of the *R.insignis* lineage. In the *R.granulata* lineage, the Asian species *R.rufobasalis* has reddish tinged stipe base, pleurocystidia with mucronate or appendiculate apices and longer terminal cells, up to 60 μm (Song et al. 2018). Finally, the typically very acrid taste allows us to eliminate most species of the *R.amoenolens* lineage, notwithstanding their sometimes quite similar colouration. The same very acrid taste also differentiates *R.obscuricolor*, which was described from the Indian Himalayas (Das et al. 2017) and showed close affinity to some Southern Hemisphere *Ingratae* in our phylogeny.

After application of these criteria, we are principally left with the phylogenetically closer species of the *R.praetervisa* lineage as potential sources of confusion, most of which are mild to merely slightly acrid. From Asia, this concerns essentially *R.pseudopectinatoides*, a species that can be distinguished by its larger basidiospores (6–) 6.5–9 (–9.5) × 5–7.5 (–8) μm, hymenial cystidia sometimes with moniliform or capitate appendages and terminal cells of pileipellis with obtuse to ventricose apices (Li et al. 2015b); *R.ahmadii* differs in small basidiomata with pileus 1–4.5 cm in diam. and pileipellis a cutis with bifurcated terminal cells (Jabeen et al. 2017). The European species *R.recondita* Melera & Ostellari has a fruity-acidic, but overall unpleasant context smell, larger basidiospores 7–8.5 × 5.5–7 μm, with ornamentation composed of mostly isolated obtuse conical warts up to 1 μm high (Melera et al. 2017). From North America, *R.amerorecondita* Avis & Barajas has a strongly tuberculate-striate pileus margin, white to pale cream spore print, larger basidiospores (6.5–) 7.1–7.6–8.1 (–9.5) × (5–) 5.6–6.3–6.9 (–8) μm with more isolated ornamentation and a habitat in hardwood forest dominated by *Quercus*; *R.garyensis* Avis & Barajas has context with unpleasant, bleachy, fishy to parmesan smell, higher basidiospore ornamentation (0.6–) 0.8–1 (–1.4) μm, longer hymenial cystidia (62–) 71.5–81.4–91 (–103) × 7–8.1–9 (–10) μm and apex sometimes with two, long, usually narrow appendages (Adamčík et al. 2019).

#### Russula succinea

Taxon classificationFungiRussulalesRussulaceae

G.J. Li & C.Y. Deng sp. nov.

741B403E-458B-51F6-8E36-BDF9AA627FEC

Fungal Names: FN 570760

**Figure 10.**
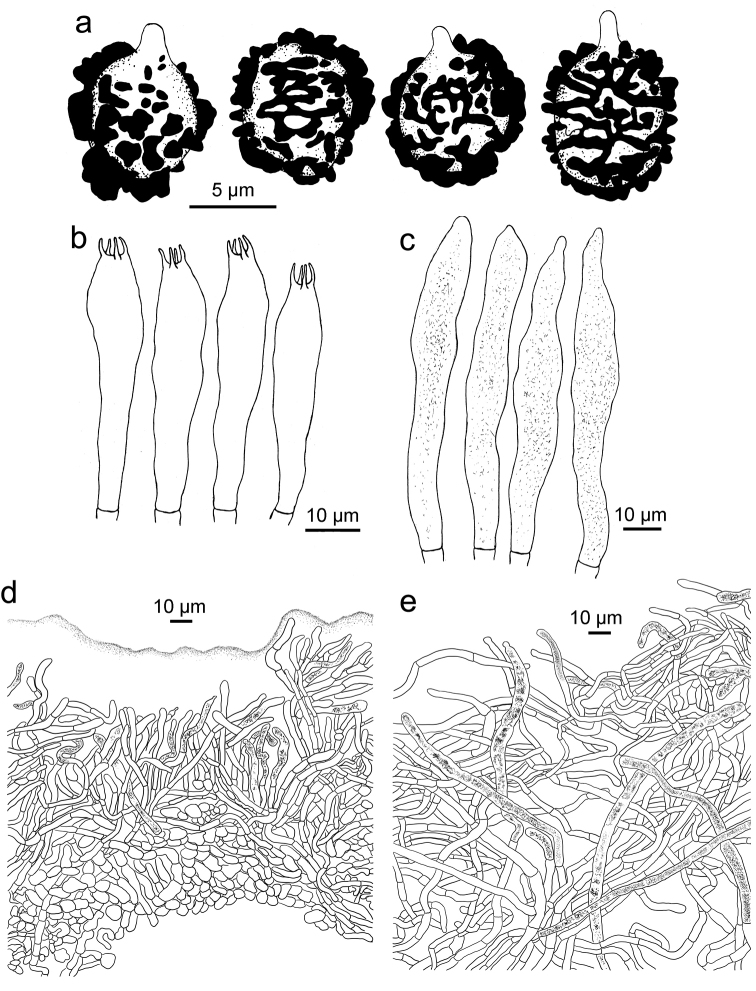
*Russulasuccinea*, holotype **A** basidiospores **B** basidia **C** hymenial cystidia**D** suprapellis partial subpellis in pileus centre **E** suprapellis in pileus margin.

**Figure 11.**
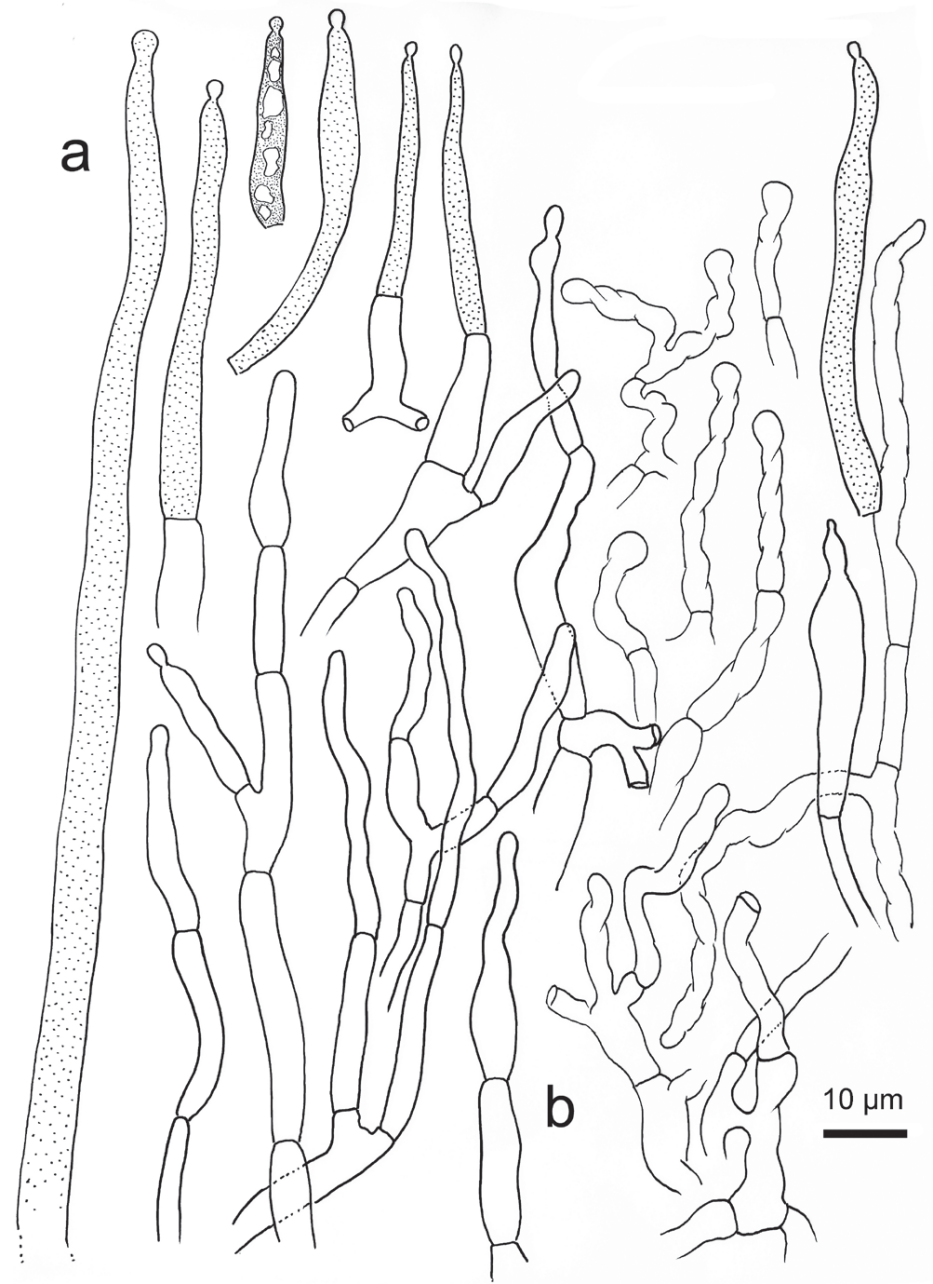
*Russulasuccinea*, holotype **A** hyphal ends in pileipellis margin **B** hyphal ends in pileus centre.

Figs 2e , 2f , 3d , 10 and 11.

##### Etymology.

referring to the pale brownish tinged pileus.

##### Holotype.

China, Guizhou Province, Weining Yi, Hui, and Miao Autonomous County, Caohai National Nature Reserve, 26°53'N, 104°12'E, alt. 2183 m, on the ground in coniferous forest, 15 July 2017, C.Y. Deng CH2017071509 (HGAS-MF 009904, **Holotype**). GenBank accession: MN649188 (ITS).

##### Diagnosis.

This species is characterised by the yellowish-brown to pale brown pileus, with tuberculate-striate margin, adnate and pale cream-coloured lamellae, subclavate to subcylindrical stipe turning cream to pale ochre when bruised, white context unchanging after injury, slightly acrid to mild taste, pale cream spore print, globose, subglobose to broadly ellipsoid basidiospores (5.8–) 6.1–7.8 (–8.3) × (4.9–) 5.2–6.8 (–7.3) μm, 7.0 × 6.0 μm on average, ornamentation 0.8–1.2 μm in height, forming incomplete reticulum, rarely intermixed with isolated warts, clavate to subcylindrical basidia, 44–66 × 10–12 μm, fusiform hymenial cystidia 71–88 × 9–15 μm, two-layered pileipellis, ixotrichodermal suprapellis in pileus centre, a trichoderm at the margin, subpellis a cutis and habitat in coniferous forests.

##### Description.

*Basidiomata* small to medium sized. *Pileus* 32–54 mm in diam., initially hemispherical, then plano-convex, flat when mature, often slightly depressed at centre, strongly viscid when wet, yellowish-brown tinged, pale brownish tinged, often intermixed with greyish-yellow fringe, Hazal (XIV11′k), Russet (XV13′k), Cinnamon Brown (XV15′k) to Tawny (XV15′) at centre, rarely with Liver Brown (XIV17′m), Pecan Brown (XXVIII13′′i) or Rood’s Brown (XXVIII11′′k) when old and dry; margin subacute to acute, straight, rarely split or inward-turned, tuberculate-striate 14–25 mm from the edge inwards, peeling 1/3–1/2 towards the centre, pale yellowish tinged, first Deep Colonial Buff (XXX21′′b), Honey Yellow (XXX19′′) to Light Ochraceous Salmon (XV13′d), then Light Cadmium (IV19), Maize Yellow (III19f) when mature. *Lamellae* adnate, 3–6 mm in height at the halfway point of pileus radius, brittle, often forked near the stipe and pileus edge, interveined, pale cream-coloured, first White (LIII), Cream Colour (XVI19′f) when mature, sometimes stained with Martius Yellow (III23f) to Baryta Yellow (IV21f); edge entire, narrowing towards the pileus margin, 13–22 pieces per cm in the edge; lamellulae absent. *Stipe* slightly subcentral, rarely central, 4.2–8.3 × 1.5–2.2 cm, subclavate to subcylindrical, often narrowing towards the base, rarely slightly curved, smooth when young, rugulose longitudinally in age, dry, Cream Colour (XVI19′f), staining Sudan Brown (III15k) to Orange-Citrine (IV19k) when bruised, Tawny Olive (XXIX17′′i), Sayal Brown (XXIX15′′) to Isabella Colour (XXX19′′i) at base, initially solid, turning hollow in age. Context White (LIII), unchanging when bruised or touched, 3–5 mm thick at the centre of pileus, fragile, taste first slightly acrid, mild when mature, odour indistinct. *Spore print* pale cream (Romagnesi IIc–IId).

*Basidiospores* [350/14/7] (5.8–) 6.1–7.8 (–8.3) × (4.9–) 5.2–6.8 (–7.3) μm, Q = (1.00–) 1.03–1.30 (–1.33) (**Q** = 1.17 ± 0.08), 7.0 × 6.0 μm on average, globose, subglobose to broadly ellipsoid, rarely ellipsoid, composed of verrucous to subcylindrical amyloid warts 0.8–1.2 μm in height, often linked as short to long crests and ridges, forming an incomplete reticulum, rarely intermixed with isolated warts; suprahilar spot distinct, but not amyloid. *Basidia* 44–66 × 10–12 μm, mostly 4-spored, clavate to subcylindrical; sterigmata 4–6 μm in length, straight to tortuous. *Hymenialcystidia* moderately numerous, ca. 700–1300/mm^2^, 71–88 × 9–15 μm, fusiform, sometimes cylindrical, thin-walled, apex obtuse, rarely mucronate, projecting 20–40 μm beyond the hymenium, contents granular to crystalline, partly dense, blackish-grey in SV. *Pileipellis* two-layered, distinctly delimited from the underlying context. The upper suprapellis (70–130 μm thick) in pileus centre an ixotrichoderm, composed of ascending to erect hyphae 4–7 μm in width, septate, cylindrical, often slightly inflated, acid-resistant encrustations absent, terminal cells sometimes narrowing towards the apex, subapical cells cylindrical, not branched; suprapellis a trichoderm in pileus margin, composed of repent, slender, cylindrical, hyaline hyphae 3–5 μm in width, acid-resistant encrustations absent. *Pileocystidia* abundant, long, cylindrical, often septate, 4–10 μm in width, apex obtuse, contents granulate, dense, blackish-grey in SV. The lower layer subpellis (50–90 μm thick) composed of loosely interwoven, mostly repent, cylindrical, septate hyaline hyphae often inflated, 2–7 μm in width. *Clamp connections* not observed in all parts.

##### Additional specimens examined.

China, Guizhou Province, Weining Yi, Hui and Miao Autonomous County, Caohai National Nature Reserve, 26°53'N, 104°12'E, alt. 2215 m, on the ground in coniferous forest, 16 July 2017, C.Y. Deng CH2017071602 (HGAS-MF 009915, paratype); ibid, alt. 2136 m, 15 July 2017, C.Y. Deng dcy2307 (HGAS-MF 009909, paratype); ibid, alt. 2005 m, C.Y. Deng dcy2309 (HGAS-MF 009906, paratype); alt. 2057 m, C.Y. Deng dcy2308 (HGAS-MF 009902, paratype); alt. 2103 m, C.Y. Deng dcy2304 (HGAS-MF 009914, paratype); Jiangxi Province, Jiujiang City, Lushan City, Lushan Mountains, alt. 1257 m, on the ground in coniferous forest, 19 October 2016, J.B. Zhang (HFJAU 0301).

##### Habit and habitat.

Single to scattered on yellow brown soil in coniferous forest dominated by *Pinusarmandii*, *P.massoniana* and *P.yunnanensis* at 1200–2200 m altitude.

##### Distribution.

China (Guizhou and Jiangxi).

##### Notes.

This new species is reminiscent of *R.foetentula*, *R.obscuricolor* and *R.rufobasalis* because of the reddish-brown or burnt sienna colour at the stipe base (Peck 1907; Song et al. 2018). The following characters are helpful for differentiating these two species from *R.succinea*: *R.foetentula* has lower basidiospore ornamentations 0.5–0.9 μm connected by occasional to rare line connections, hymenial cystidia with mucronate-appendiculate apices 2–7 μm long, pileocystidium apex often constricted to 1–2.5 μm in width; North American distribution (Peck 1907; Adamčík et al. 2013); *R.obscuricolor* has darker brown to chocolate brown tinges at pileus centre, bitter to pungent taste of context and shorter hymenial cystidia (pleurocystidia 30–65 × 6–9 μm, cheilocystidia 23–33 × 5–7 μm) (Das et al. 2017); *R.rufobasalis* has bright reddish tinge at stipe base, basidiospore ornamentations 0.3–0.8 μm in height and frequently thick-walled, narrower terminal cells 2–5 μm in width (Song et al. 2018).

For those Asian sect. Ingratae members that have similar pileus tinges, *R.ahmadii* can be distinguished from *R.succinea* by lower basidiospore ornamentations up to 0.3 μm, shorter basidia (29–) 29.7–38.9 (–40.1) × (9.2–) 9.4–11.3 (–11.8) μm and pileipellis a cutis (Jabeen et al. 2017); *R.arunii* differs from the new species in the orange tinge intermixed on pileus surfaces, white spore print, narrow pileocystidia 3–4 μm in width and a habit of broad-leaved *Pterygotaalata* forest (Crous et al. 2017); *R.catillus* differs in that basidiospore ornamentation is composed of mostly isolated, verrucous to conical warts, absence of pileocystidia in pileipellis and a habitat of oak hardwood forest (Lee et al. 2017); *R.indocatillus* can be differentiated from the new species for white spore print, shorter basidia 34–40 × 9–11 μm and capitate hymenial cystidium apex (Ghosh et al. 2020); *R.natarajanii* differs in having light to medium brown spots at the pileus periphery, shorter basidia 28–35 × 7.5–9 μm and a habitat of *Quercus* forest (Das et al. 2006); *R.pseudocatillus* differs in the presence of lamellula, basidiospores ornamented with isolated warts never forming a reticulum and a habitat of broad-leaved evergreen forest (Yuan et al. 2019); *R.pseudopectinatoides* can be distinguished from the new species in having hymenial cystidia with moniliform or capitate apex, larger basidiospores up to 9 μm in diam. and absence of pileocystidia (Li et al. 2015b); *R.straminella* differs in its shorter basidia and hymenial cystidia, often thick-walled terminal cells in pileipellis of pileus margin (Figs 6 and 7); *R.gelatinosa*, *R.punctipes*, *R.seneicis*, *R.subpunctipes* and *R.tsokae* differ from *R.succinea* in their larger basidiospores (9 μm in diam.) with high ornamentation up to 2 μm in height (Khatua et al. 2015; Lee et al. 2017; Song et al. 2018; 2020).

## Discussion

The modern taxonomy of *Russula* calls for a combination of detailed microscopic observations with universal and specific standard, multi-gene phylogenetic analyses and accurate symbiotic plant species information (Buyck et al. 2018; Adamčík et al. 2019). The ITS phylogenetic analyses are the most common for practical identification of *Russula* species, because ITS is regarded as an adequate single gene DNA barcode for this genus (Li et al. 2019) and it has the largest number of available referential sequences in open databases (Schoch et al. 2012). A combination of morphological and ITS phylogenetic analyses supported the three new species amongst Asian *Ingratae*: *R.straminella*, *R.subpectinatoides* and *R.succinea*. The results of this study also indicate that *R.indocatillus* may have a wider distribution, from the Himalayan region to south-western China. The four species discussed here have distinct morphologies that allow each one to be differentiated from the others:

- R. subpectinatoides and R. indocatillus possess the more or less inflated, short-celled chains of hyphal ends, typical for most species in the subgenus Heterophyllidiae (Figs 4, 5, 8 and 9). These are abundant in R. subpectinatoides, but less so in R. indocatillus and absent in both other species which possess very dense, intricate and strongly branching, narrow ends in the pileipellis, more or less cemented in mucus that make microscopic examination of these hyphal ends very difficult. Compared to R. straminella, hyphal ends in the pileus centre of R. succinea have a more wavy-undulate form (Figs 6, 7, 10 and 11).

All four species have similar pileocystidia, but in *R.indocatillus*, they are smaller overall at the pileus surface compared to the other three species (Figs 4 and 5), while in *R.straminella*, they are often more or less thick-walled (Figs 6 and 7).

When comparing basidiospores, *R.subpectinatoides* stands out because of the low subreticulate ornamentation (Figs 3 and 8), whereas the other species have more developed, higher warts or ridges that are much less interconnected, while *R.indocatillus* has almost completely isolated warts (Figs 3 and 4).

Some European members of section Ingratae, viz. *R.amoenolens* Romagn., *R.pectinata* Fr., *R.pectinatoides* Peck and *R.sororia* (Fr.) Romell may have been confused morphologically with some of these new species (Wu 1989; Ying and Zang 1994), but more recent diversity analyses indicated that some Chinese specimens, identified as *R.amoenolens* and *R.insignis* Quél., have broad morphological similarities, but also considerable difference (ca. 2%) in the ITS sequence compared to European samples of these species (Li 2014; Liu et al. 2017; Cao et al. 2019). Whether these Chinese specimens represent unknown taxa or intraspecific geographically-separated populations is still debatable (Wang 2020). The factual presence of these species of European and North American origin in China have been analysed in recent years (Li 2014; Zhang 2014; Wang 2019; Liu 2019) and symbiotic host plants were found to be very similar between north-eastern China, Europe and North America (Wu 1979).

The topology of the ITS phylogram (Fig. 1) in this study largely corresponds to that of Park et al. (2017). Of the three subsections in sect. Ingratae, the majority of subsect. Pectinatinae Bon (type species *R.pectinata*) with species that are typically more greyish-brown to greyish-cream is distributed over clades C and H (Bon 1988), while *Subvelatae* (Singer) Singer (type species *R.subvelata* Singer) with members that have velar rudiments consisting of loosely, arachnoid-pulverulent floccons on pileus surface (Singer 1986), forms the highly-supported clade I. The species *R.indocatillus*, newly-recorded from China in this study, is located in Clade H. This well-supported clade also contains the *R.amoenolens* complex from Europe and *R.cerolens* and allies from North America. The African species complex of *R.oleifera* Buyck in subsect. Oleiferinae Buyck (type species *R.oleifera* Buyck) with species that sometimes present an annulus, corresponds to Clade D (Sanon et al. 2014). This clade was a sister clade to the remainder of sect. Ingratae in the multilocus phylogenetic analysis of Buyck et al. (2018). The large majority of European species that cluster around *R.foetens* compose clade F, a clade highly supported by Bayesian analysis only. The latter clade is typically composed of yellowish-brown to orange brown species and roughly corresponds to species traditionally placed in subsect. Foetentinae (Melzer & Zvara) Singer (type species *R.foetens*), of which it is characterised by dull, ochraceous or pallid coloured pileus, often with pectinate-sulcate to tuberculate-sulcate and distinctly subacute to acute margin, context odour of nitrobenzene, oily, fish, iodoform, or of other unpleasant smells (Singer 1986). Clade F also contains two of our new species, *R.straminella* and *R.succinea*, which share a similar pileipellis structure. Clade E received higher support in ML and MP analyses and shared with Clade F that two of the three species were also yellowish- to orange brown. This clade harbours three species: *R.rufobasalis* from Asia and the North American *R.granulata* Peck and *R.ventricosipes*. The results of our phylogenetic analyses, based on ITS sequences, indicate that more unknown subsections may exist in sect. Ingratae. More complex multi-gene analyses are urgently needed to clarify the phylogenetic relationships amongst species in this section.

Compared with previous analyses (Melera et al. 2016; Lee et al. 2017), more gasteroid species of sect. Ingratae were included in our study. The majority of gasteroid taxa clustered as two branches in Clade F. The other gasteroid species were mainly scattered in clades of agaricoid taxa. The phylogenetic topologies and low supported branches within sequestrated complex 2 may indicate an urgent need to study the type material of these gasteroid species for clarification of synonyms.

Lee et al. (2017) summarised the general patterns observed for spores in the four clades of sect. Ingratae by showing a trend for basidiospore size to increase, while the shape changes from ellipsoid to spherical and for species that have smaller spores to have more ellipsoid spores and vice versa. However, these patterns were less clear when gasteroid species of this section were taken into account (Table 2). These gasteroid species suggest that the patterns, proposed in Lee et al. (2017), do not fit well with all members of the sect. Ingratae. Gasteroid taxa are known to have typically more globose and larger spores, because there are no evolutionary pressures of asymmetrical spores with hilar appendages for ballistospory in agaricoid species (Wilson et al. 2011). According to statistics, exceptions that do not follow these general patterns are common in sect. Ingratae. Over 40% (5/11) of counted gasteroid species of this section have subglobose to broadly ellipsoid, even ellipsoid spores. In simple terms, a significant portion of gasteroid species have larger, but still more ellipsoidal spores. The authors suggested that these exceptions may be ascribed to the multiple and irreversible evolutions of gasteromycetation (Miller et al. 2002; Hibbett 2007). Ancestor genotype, divergence time and environmental factors all may exert different influences on this phenotype.

**Table 2.** Spore sizes and shapes of gasteroid sect. Ingratae species.

Species	Spore size (μm)	Spore shape (Q value)	Reference
*R.ammophila* (J.M. Vidal & Calonge) Trappe & T.F. Elliott	7–9 × 5.5–7.5	subglobose to broadly ellipsoid	Vidal et al. (2002)
*Russulaaromatica* Trappe & T.F. Elliott	8–11 × 7.5–10	globose to subglobose	Smith (1963)
*R.brunneonigra* T.Lebel	11–14(–15) × 11–13(–15)	globose (Q = 1.00–1.03)	Lebel and Tonkin (2007)
*R.galbana* T.Lebel	8–10 × 8–10	globose (Q = 1.01–1.06)	Lebel and Tonkin (2007)
*R.mistiformis* (Mattir.) Trappe & T.F. Elliott	(8.5–) 9.5–11 (–12.5) × (8–) 8.5–10 (–10.5)	subglobose to broadly ellipsoid (Q = 1.1–1.2)	Vidal et al. (2019)
*R.nondistincta* (Trappe & Castellano) Trappe & T.F. Elliott	7–11 in diam.	globose	Trappe and Castellano (2000)
*R.parksii* (Singer & A.H. Sm.) Trappe & T.F. Elliott	8–11 × 7–9 /10–14(–18) × 9–12(–14)	subglobose to ellipsoid	Singer and Smith (1960)
*R.pilosella* (Cribb) T.Lebel	8.5–10 × 8–9.5	subglobose to broadly ellipsoid (Q = 1.07–1.2)	Lebel and Tonkin (2007)
*R.similaris* Trappe & T.F. Elliott	9–12 × 8–10	globose to subglobose	Singer and Smith (1960)
*Russulashafferi* Trappe & T.F. Elliott	8–11 × 8–9	subglobose to broadly ellipsoid	Singer and Smith (1960)
*Russulasubfulva* (Singer & A.H. Sm.) Trappe & T.F. Elliott	9–12 × 8–11	globose to subglobose	Singer and Smith (1960)

Spore ornamentations consisting of winged ridges are regarded as one of the most distinctive morphological characters for some members of sect. Ingratae. These species include *R.grata*, *R.fragrantissima* and *R.illota* from Europe and northern China, *R.mutabilis* from North America, *R.gelatinosa*, *R.punctipes*, *R.subpunctipes* and *R.senecis* from eastern and southern Asia. A majority of these species and *R.foetens* formed a not highly supported clade in phylogenetic analyses of Lee et al. (2017). As more samplings and species of sect. Ingratae were involved, the monophyly of winged-spore species was not supported in this analysis. Close phylogenetic relationships were detected in strongly-supported clades of *R.grata*-*R.fragrantissima*, *R.mutabilis*-*R.illota* and *R.punctipes*-*R.subpunctipes*-*R.senecis*. This phylogenetic inconsistency called for a further multi-gene analysis.

The habitats of the four species of this study show a common feature of coniferous forests dominated by *Pinus* spp. The current altitudes of distributions of *R.indocatillus* and *R.succinea* indicate a habitat of subalpine climate. These two species may have wider distributions than current records because the corresponding ectomycorrhizal symbiotic trees are representative and widespread species in Sino-Japanese and Sino-Himalayan floral subregions (Wu 1980; Chen et al. 2020). For *R.straminella* and *R.subpectinatoides* which were collected from reforested plantations and transplanted botanic gardens, intensive samplings on initial areas of symbiotic trees are needed for clarifying the types of habitats.

Specimens of the four species in this analysis were all collected on yellow brown soil. Local analyses showed high nitrogen conditions in soil environments of these species (Cai et al. 2010; Wang et al. 2010; Zhang et al. 2014). This result supported the conclusions in Avis (2012) that nitrophilic tendencies appear throughout fetid Russulas.

## Supplementary Material

XML Treatment for Russula indocatillus

XML Treatment for Russula straminella

XML Treatment for Russula subpectinatoides

XML Treatment for Russula succinea

## Supplementary materials

Supplementary material 1 Fasta file for phylogenetic analyses Data type phylogenetic data https://binary.pensoft.net/file/608658This dataset is made available under the Open Database License (http://opendatacommons.org/licenses/odbl/1.0/). The Open Database License (ODbL) is a license agreement intended to allow users to freely share, modify, and use this Dataset while maintaining this same freedom for others, provided that the original source and author(s) are credited.Guo-Jie Li

Supplementary material 2 Phylip file for ML analysis Data type phylogenetic https://binary.pensoft.net/file/608659This dataset is made available under the Open Database License (http://opendatacommons.org/licenses/odbl/1.0/). The Open Database License (ODbL) is a license agreement intended to allow users to freely share, modify, and use this Dataset while maintaining this same freedom for others, provided that the original source and author(s) are credited.Guo-Jie Li

Supplementary material 3 Nexus file for Baysian analysis Data type phylogenetic https://binary.pensoft.net/file/608660This dataset is made available under the Open Database License (http://opendatacommons.org/licenses/odbl/1.0/). The Open Database License (ODbL) is a license agreement intended to allow users to freely share, modify, and use this Dataset while maintaining this same freedom for others, provided that the original source and author(s) are credited.Guo-Jie Li

Supplementary material 4 Nexus file for MP analysis Data type phylogenetic https://binary.pensoft.net/file/608661This dataset is made available under the Open Database License (http://opendatacommons.org/licenses/odbl/1.0/). The Open Database License (ODbL) is a license agreement intended to allow users to freely share, modify, and use this Dataset while maintaining this same freedom for others, provided that the original source and author(s) are credited.Guo-Jie Li
